# Physicochemical Factors Influence the Abundance and Culturability of Human Enteric Pathogens and Fecal Indicator Organisms in Estuarine Water and Sediment

**DOI:** 10.3389/fmicb.2017.01996

**Published:** 2017-10-17

**Authors:** Francis Hassard, Anthony Andrews, Davey L. Jones, Louise Parsons, Vera Jones, Brian A. Cox, Peter Daldorph, Howard Brett, James E. McDonald, Shelagh K. Malham

**Affiliations:** ^1^School of Ocean Sciences, Bangor University, Bangor, United Kingdom; ^2^Cranfield Water Science Institute, Cranfield University, Bedford, United Kingdom; ^3^UK Water Industry Research Limited, London, United Kingdom; ^4^School of Environment, Natural Resources and Geography, Bangor University, Bangor, United Kingdom; ^5^Atkins Limited, Bristol, United Kingdom; ^6^Atkins Limited, Warrington, United Kingdom; ^7^Atkins Limited, Peterborough, United Kingdom; ^8^Thames Water Utilities, Reading, United Kingdom; ^9^School of Biological Sciences, Bangor University, Bangor, United Kingdom

**Keywords:** sediment, fecal indicator organisms, pathogen, elemental analysis, viable but non-culturable bacteria

## Abstract

To assess fecal pollution in coastal waters, current monitoring is reliant on culture-based enumeration of bacterial indicators, which does not account for the presence of viable but non-culturable or sediment-associated micro-organisms, preventing effective quantitative microbial risk assessment (QMRA). Seasonal variability in viable but non-culturable or sediment-associated bacteria challenge the use of fecal indicator organisms (FIOs) for water monitoring. We evaluated seasonal changes in FIOs and human enteric pathogen abundance in water and sediments from the Ribble and Conwy estuaries in the UK. Sediments possessed greater bacterial abundance than the overlying water column, however, key pathogenic species (*Shigella* spp., *Campylobacter jejuni, Salmonella* spp., hepatitis A virus, hepatitis E virus and norovirus GI and GII) were not detected in sediments. *Salmonella* was detected in low levels in the Conwy water in spring/summer and norovirus GII was detected in the Ribble water in winter. The abundance of *E. coli* and *Enterococcus* spp. quantified by culture-based methods, rarely matched the abundance of these species when measured by qPCR. The discrepancy between these methods was greatest in winter at both estuaries, due to low CFU's, coupled with higher gene copies (GC). Temperature accounted for 60% the variability in bacterial abundance in water in autumn, whilst in winter salinity explained 15% of the variance. Relationships between bacterial indicators/pathogens and physicochemical variables were inconsistent in sediments, no single indicator adequately described occurrence of all bacterial indicators/pathogens. However, important variables included grain size, porosity, clay content and concentrations of Zn, K, and Al. Sediments with greater organic matter content and lower porosity harbored a greater proportion of non-culturable bacteria (including dead cells and extracellular DNA) in winter. Here, we show the link between physicochemical variables and season which govern culturability of human enteric pathogens and FIOs. Therefore, knowledge of these factors is critical for accurate microbial risk assessment. Future water quality management strategies could be improved through monitoring sediment-associated bacteria and non-culturable bacteria. This could facilitate source apportionment of human enteric pathogens and FIOs and direct remedial action to improve water quality.

## Introduction

Human exposure to pathogenic microorganisms through contact with polluted recreational waters or ingestion of contaminated seafood is a serious public health concern, resulting in ~2.5 million deaths, 250 million cases of gastrointestinal infection and 4 million lost person-years annually (Kay et al., 2005; Stapleton et al., 2007; Gao et al., 2013; Moore et al., 2013; Henry et al., 2016a,b). Coastal and estuarine regions typically have a high population density, discharging wastewater and agricultural/urban runoff into waters used for recreation and shellfish aquaculture (Malham et al., 2014). The magnitude of surface runoff is projected to increase under future climate scenarios (Semenza and Menne, 2009). Apportioning the source of human enteric pathogens and FIOs within coastal and estuarine environments is challenging due to the complex and transient nature of inputs, which can be direct or diffuse, and may contain fecal matter. Sources include agricultural run-off, combined sewer overflows (CSOs), wastewater treatment works (WwTWs), wild animals and autochthonous inputs. The disease risk is typically proportional to the dose of pathogenic microorganisms ingested or inhaled (Atmar et al., 2014). As pathogenic microograngisms have low abundance and are usually transient in the environment, the measurement of these organisms is costly, time consuming and requires specialist equipment and personnel (Gao et al., 2013; Perkins et al., 2014). The EU Water Framework Directive has set standards for levels of fecal indicator organisms (FIOs) for safe recreational/shellfish water quality. FIOs are assessed to minimize the risk to the public, and this is achieved through monitoring and management practices set by regulatory agencies (e.g., EC 2006/7/EC), (EC, 2006). Target organisms for culture-dependent quantification on selective microbiological media include *Escherichia coli* and intestinal enterococcus, with FIO abundance typically correlating with the amount of fecal pollution. However, the veracity of FIOs as an indicator of fecal pollution is dependent upon the assumption that sites free of fecal contamination do not contain these indicators (e.g., Thomann and Muller, 1987). Sediments can be long term reservoirs of FIOs within the environment, and therefore, the widespread use of bacterial indicators to assess water quality is being challenged (Anderson et al., 2005). The persistence of different strains/species of indicator appears strongly influenced by physicochemical factors in the environment, which is of concern in dynamic coastal and estuarine environments. Autochthonous growth of FIOs can also occur in natural waters, presenting a challenge to the use of FIOs for quantitative microbial risk assessment (QMRA; Anderson et al., 2005). Furthermore, some bacteria have been shown to exist in a viable but non-culturable state (alive but not cultivatable on microbiological growth media). Viable but non-culturable bacteria cannot be detected by conventional microbiological culture-based methods, but retain the ability to resuscitate under suitable conditions and could therefore present a risk to human health (Bonilla et al., 2007; Yamahara et al., 2007; Zakhour et al., 2010; Vignaroli et al., 2013; Pinto et al., 2015). Molecular methods such as quantitative (qPCR) and reverse transcription qPCR (RT-qPCR) are often used for the rapid and accurate enumeration of pathogen-derived nucleic acids in environmental samples (Farkas et al., 2017). Quantitative PCR methods represent a suitable method for maximal bacterial or viral recovery from water or sediment (including non-culturable bacteria), however, few studies have undertaken side-by-side comparison of quantitative PCR and plate count methods.

An additional concern in water quality monitoring is that bacterial indicators may misrepresent the risk from enteric viruses, which account for most gastrointestinal infections from bathing waters and shellfish (Baert et al., 2011; Rusiñol et al., 2014). The persistence of FIOs in waters has been well studied, notably the negative correlation between salinity and *E. coli* persistence (Anderson et al., 1979) and positive association with organic material, nutrients, clay content and suspended particulate matter (LaBelle and Gerba, 1979). Furthermore, seasonal and diurnal variation in the abundance of FIOs has been shown to vary with water temperature and light intensity (Kay et al., 2005; Mudd et al., 2012). However, the physicochemical factors which govern survival/persistence in sediments has received much less attention to date (Gerba and McLeod, 1976; Perkins et al., 2014), despite bacterial abundance in sediments being up to 10,000-fold greater than in the overlying water column (Bai and Lung, 2005). Sediments represent a poorly monitored risk factor for water quality, however, it is likely that resuspension of fine sediments and organic matter can significantly increase waterborne bacterial loads (Jamieson et al., 2005; Pachepsky and Shelton, 2011; Drummond et al., 2014). Despite this, bacterial standards and monitoring regimes are yet to be implemented for sediments by most regulatory agencies (Solo-Gabriele et al., 2000, 2015; Vignaroli et al., 2013). Sediment grain size, organic matter content, porosity and mineral composition (and chemical species within) have all been linked to the abundance and distribution of fecal bacteria (Pachepsky and Shelton, 2011; Cai et al., 2013; Perkins et al., 2014). The impact of sediment chemical species on the viability/or culturability of bacteria during adhesion has been established in pure culture (e.g., Gottenbos et al., 2001). However, the impact of sediment chemical species on bacterial indicator/pathogen ecology (including FIOs) has yet to be demonstrated in the field within coastal and estuarine environments. As a general hypothesis, we postulate that the presence and magnitude (linked to sources and persistence) of bacterial contamination within coastal and estuarine environments (i.e., water and sediments) will depend on a range of physicochemical factors, notably; salinity, temperature, sediment organic matter and sediment grain size. The objective of this study was to assess the physicochemical factors which influence the abundance and culturability of selected human enteric pathogens and fecal indicator organisms in estuarine water and sediment.

## Materials and methods

### Study locations

**Figure 1:**
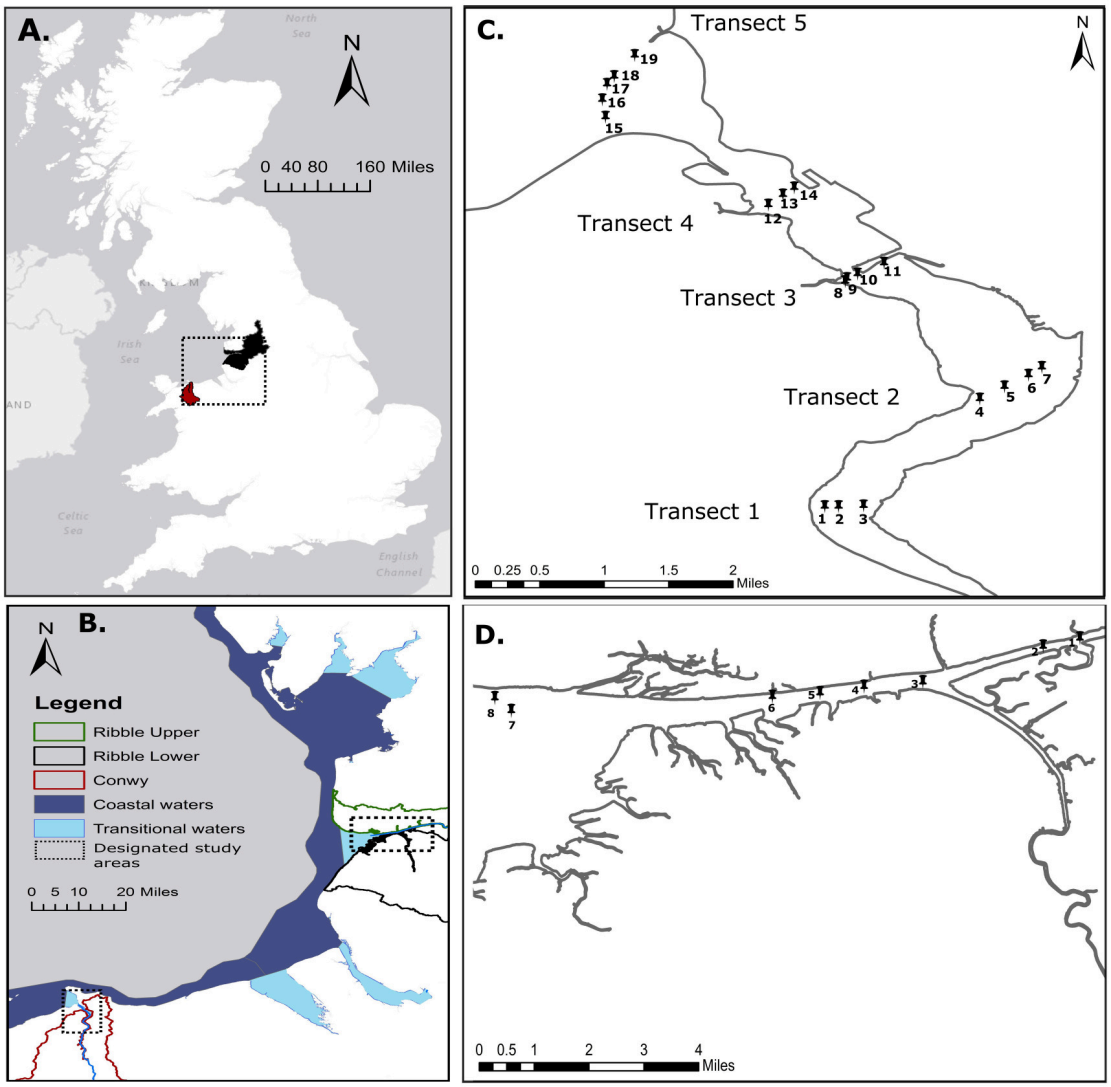
Study locations. **(A)** Catchment scale Conwy, Wales, UK and Ribble, England, UK. **(B)** North West UK coastal and transitional waters are shown (2006/7/EC) and designated estuarine study locations (black box) are indicated. **(C)** Conwy Estuary with five transverse transects with water and sediment collected at nineteen samples sites. **(D)** Ribble Estuary with water and sediment collected at eighth sample sites. Conwy catchment and Ribble Upper and Lower catchments are shown.

**Figure 2:**
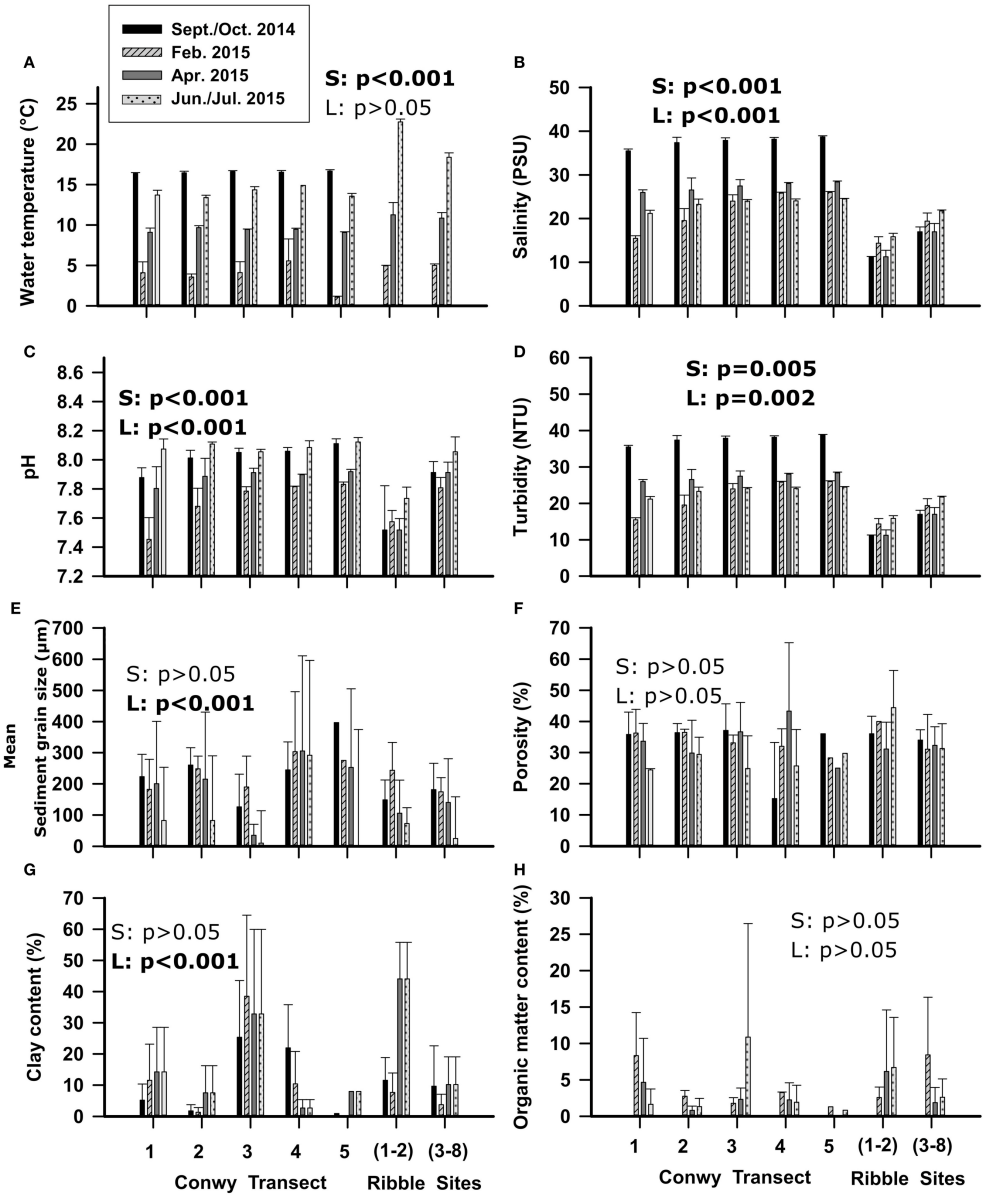
Physicochemical variables of the water **(A–D)** and sediment **(E–H)** of the Conwy estuary from south (transect 1) to north (transect 5) and Ribble estuary from east (sites 1–2) to west (sites 3–8). Mean ± standard deviation. Statistical results of Welch's *t*-test are presented within graphs, with S, effect of season; L, effect of location (Conwy transects and Ribble sites). Significant results are shown in bold.

We selected two study areas: (1) the Conwy estuary located in North Wales, UK (Figures 1A,B), and (2) the Ribble estuary located in North West England, UK (Figures 1A,B). Sample sites in the Conwy estuary were selected to represent possible point and diffuse sources of pollution that include agricultural land predominantly utilized for livestock grazing (transect 1), mudflats used by wading birds and agricultural land (transect 2), input from an urban area via the River Gyffin (transect 3), and input from a commercial marina (transect 4), and a EC 2006/7/EC coastal/transitional waters boundary (transect 5) (Figure 1C). Sample sites in the Ribble estuary were selected to represent possible point and diffuse sources of pollution for wastewater discharges (sites 1 and 2), confluences with other tributaries (sites 3 and 4), wading birds and agricultural land (sites 5 and 6) and urban areas (sites 7 and 8). Due to the complex mosaic of land uses in each catchment, a mixture of pollution inputs were expected at most sampling sites, however, they were chosen based on the dominant land use.

The Conwy estuary discharges into the Irish Sea (Figure 1B) with annual rainfall across the 342 km^2^ catchment ranging from 500 mm close to the coast to 3,500 mm in the upper reaches (Emmett et al., 2016). The estuary receives inputs from a catchment dominated by agriculture and forestry. The Conwy catchment has an average population density of 304 persons km^−2^. One of the main wastewater inputs is the Ganol WwTW (~82,000 population equivalent; PE) which discharges disinfected secondary effluent through a sea outfall to the east of Llandudno into Penrhyn Bay. The Conwy has a further 19 WwTW discharging secondary treated wastewater with a total PE of 15,000 PE (mean = 791, range = 38–4,987 PE) (DCWW, 2015). The estuary also receives intermittent CSO discharges, during peak run-off. The Ribble estuary receives inputs from an area of mixed land use with a total area of ~1,580 km^2^ with rainfall ranging from 84 to 1,520 mm per year (30-year average 1961–1990 data from the UK Met Office, Station ID 71001, Ribble at Samlesbury). The Ribble catchment has an average population density of 1,580 persons km^−2^. Preston Clifton Marsh WwTW (>100,000 PE) discharges tertiary treated effluent (UV disinfected) into the River Ribble. 48 other WwTW and at least 24 CSOs discharge intermittently directly or indirectly into the Ribble estuary.

### Estuarine sampling

Estuarine sediment and water samples were collected from four transverse transects of the Conwy estuary with 19 sample sites, over four sampling days (Event 1C = 29.9.2014, Event 2C = 3.2.2015, Event 3C = 24.04.2015, Event 4C = 17.06.2015). Sampling in the Ribble was undertaken from east to west over four sampling days (Event 1R = 27.10.2014, Event 2R = 9.02.2015, Event 3R = 22.04.2015, Event 4R = 8.7.2015) (Figure 1D). Henceforth, these sampling events are designated by month September/October (Event 1), February (Event 2), April (Event 3), June/July (Event 4). Samples were taken by boat at high tide. Water samples were collected in triplicate from 0.2 m below the water surface for each sampling point using sterile 1 l polypropylene containers, prior to collecting sediment. For each sampling point, three replicate sediment samples were collected using a manually operated Van Veen sediment grab; ~50 g of bottom sediment was taken from the center of each grab and transferred into sterile 50 ml polypropylene tubes (VWR International Ltd., Leicestershire, UK). All sampling equipment was rinsed with deionized water and site water prior to use between sampling sites. Water and sediment samples were transported to the laboratory in chilled containers and processed within the 6 h recommended window from first sample collection (2006/7/EC). Subsequently, sediment aliquots (~1 g) were taken from each replicate grab and frozen at −80°C pending nucleic acid extraction. For bacterial genetic analysis of water, duplicate water samples were filtered (ca. 500 ml) through 0.2 μm filters (Sartorius, Germany). For viral analysis in water, 1 l subsamples were taken, subjected to acidification in the presence of a cation and filtered through a negatively charged filter following a modified method from Katayama et al. (2002). Briefly MgCl_2_ was added to a final concentration of 25 mM, then the pH was adjusted to 3–5 units using 1 M HCl, then samples were subsequently shaken at 200 rpm for 30 min. Samples were pre-filtered using 1.2 μm glass fiber filters (Whatman, UK). Water was subsequently filtered through negatively charged 0.45 and 0.22 μm cellulose nitrate filters (Sartorius, Germany). Filters were rinsed with 0.8 M NaCl. The pre-filters were discarded and the 0.45 and 0.22 μm containing the DNA and RNA viruses were frozen at −80°C prior to further analysis.

### Isolation and cultivation of target bacterial groups from sediment

Isolation and cultivation approaches followed those described in Perkins et al. (2014). Briefly, 1 g of each sediment sample was transferred to a 7 ml sterile bijou tube (Starlab UK Ltd., Milton Keynes, UK) and suspended in 5 ml Ringers solution (Oxoid Ltd., Basingstoke, UK) to obtain a 1:5 (w/v) dilution. Each sample was vortexed for 180 s and aliquots of the resulting supernatant for each sample were transferred aseptically onto agar plates containing a selective medium for *E. coli* and coliforms (Harlequin *E. coli*/Coliforms Media, Lab M, UK), intestinal enterococci (Slanetz and Bartley Media, Lab M, UK), *Vibrio* spp. (TCBS media, Oxoid, UK), non-specific minimal medium for total heterotrophic bacteria (R2A, Oxoid, UK) and total marine heterotrophic bacteria (Marine Agar, Deben Diagnostics Ltd., UK). The optimum volume of supernatant used to inoculate each microbiological plate was determined previously (Perkins et al., 2014). Incubation temperatures/times were controlled and were identical between sites/sampling events (Table S1). Resulting colony forming units (CFUs) per 100 ml of water or per 100 g sediment provided an estimate of culturable bacterial numbers. The detection limit for culturable bacteria was 1 CFU g^−1^ for sediment samples.

### Isolation and cultivation of target bacterial groups from water

Enumeration of bacteria in water samples was achieved by using vacuum–filtration (Quilliam et al., 2011). Briefly, water samples were homogenized by shaking, a log_10_ dilution series was undertaken and 50 ml of water was filtered under vacuum through a 0.2 μm cellulose nitrate membrane (Sartorius, Germany). Subsequently, the membranes were aseptically transferred onto sterile agar plates containing selective medium for the enumeration of *E. coli*, coliforms and intestinal enterococcus. Averages of appropriate dilutions were calculated (ISO 9308-1, 1990). For *Vibrio* (presumptive), heterotrophic bacteria and marine heterotrophic bacteria log dilutions were undertaken with ¼-strength ringers, 200 μl was then drop-plated and spread aseptically. Averages of appropriate dilutions were calculated (ISO 9308-1, 1990). Plates were incubated and enumerated as described above. The detection limits for culturable bacteria was 1 CFU/100 ml for water samples.

### Nucleic acid extraction

Nucleic acids were extracted directly from water using the method of Griffiths et al. (2000) with minor modifications. Filters (separately for bacteria and virus) were removed from the −80°C freezer and immediately placed in 0.5 ml of warmed hexadecyltrimethylammonium bromide (CTAB) extraction buffer and 0.5 ml of phenol-chloroform-isoamyl alcohol (25:24:1) (pH 8.0) (PCI) with 0.5 g glass beads, followed by mechanical lysis at 5.5 m s^−1^ for 30 s in a ribolyzer (Hybaid). We used the protocol outlined in Chiao et al. (2014) which involves a 70°C incubation step with PCI to allow for the disintegration of the filter used to collect microbial cells, to ensure minimal interference of the filter itself during the bead-beating step. Samples were centrifuged (14,000 × g, 5 min) and the top aqueous phase (containing nucleic acids) was transferred to a new tube. Equal volumes of chloroform-isoamyl alcohol (24:1) (CI) were added, followed by centrifugation (14,000 × g, 5 min). Nucleic acids were precipitated from the aqueous layer by the addition of 2 volumes of 30% (wt/vol) polyethylene glycol 6000 (PEG6000)—1.6 M NaCl. The mixture was incubated at room temperature for 2 h and centrifuged at 16,000 × g for 10 min. The pellet was washed with 0.2 ml ice-cold 70% ethanol and air dried prior to elution in 50 μl molecular-grade water. The concentration and quality of genomic DNA was determined using a Qubit fluorimeter 2.0 (Invitrogen, UK) and a Nanodrop ND-1000 (Nanodrop, USA), respectively. Results suggested high dsDNA concentrations in water samples. However, the Griffiths protocol resulted in poor quality and concentration of nucleic acids for sediment (< 0.02 ng) therefore a Powersoil DNA extraction kit (MO BIO Laboratories, USA) was used for DNA extraction from 0.25 g of sediment per the manufacturer's instructions and eluted in 50 μl molecular-grade water. Subsequent analysis suggested high dsDNA concentrations in sediment samples. For RNA viruses, a Powersoil RNA extraction kit (MO BIO Laboratories, USA) was used for RNA extraction from 2 g of sediment per manufacturer's instructions and eluted in 50 μl molecular-grade water. All samples were analyzed using qPCR and RT-qPCR as described below.

### Quantitative PCR

All qPCR assays were carried out in a QuantStudio™ Flex 6 Real-Time PCR System (Applied Biosystems, USA). For quantification, a dilution series of a plasmid DNA carrying the target sequence were used with a dynamic range of 2–2 × 10^6^, qPCR assays with standard curves *R*^2^ values < 0.99 were rerun (Primerdesign Ltd., UK). For all samples, the original and a ten-times diluted extract of DNA or RNA were tested. Standards, primers and probes were obtained from commercially available kits (Genesig, Primerdesign Ltd., UK). Bacterial targets specific for *E. coli, Salmonella* spp., *Campylobacter jejuni, Enterococcus faecium, Enterococcus faecalis, Vibrio* spp. and *Shigella* spp. were selected (Table S2). A TaqMan-based (hydrolysis probes) qPCR was undertaken with the Oasig q-PCR Master Mix (Primerdesign Ltd., UK), according to the manufacturer's instructions on three replicate samples. The 20 μl reaction mixes contained 1x qPCR mix, 1 μg bovine serum albumin (BSA), 1 μl Primer/Probe mix and 4 μl sample or standard (Primerdesign Ltd., UK). Initial denaturation was for 2-min denaturation at 95°C and 50 cycles of amplification consisted of 95°C for 15 s and 60°C for 60 s. Viral targets specific for norovirus GI, norovirus GII, hepatitis A virus, hepatitis E virus were selected (Table S2). RT-qPCR was undertaken with the Oasig OneStep RTqPCR Master Mix (Primerdesign Ltd., UK). For RT-qPCR assays extracted samples were normalized to a final concentration of 5 ng/μl of sample RNA (Bustin et al., 2009). The 20 μl reaction mixes contained 1x RT-qPCR mix, 1 μg bovine serum albumin (BSA), 1 μl Primer/Probe mix and 4 μl sample or standard (Primerdesign Ltd., UK). The reverse transcription was performed at 42°C for 10 min followed by a 2-min at 95°C and 50 cycles of amplification consisted of 95°C for 15 s and 60°C for 60 s. The absolute detection limits for each target was assumed as 1 Gene Copy (GC)/g for sediment samples and 1 CFU/100 mL for water samples. In practice, values should be treated with caution due to the accuracy of absolute quantification at levels <100 GC/cm^3^ of water or sediment based on comparison with standard curves. Extraction efficiency was assessed in sediments from 10% of sediment and water samples through spiking with plasmid DNA and qPCR/RT-qPCR efficiency/inhibition were assessed using plasmid DNA external controls and found to be between 1 and 10% efficiency. Retesting occurred due to failed PCR controls or qPCR standard curve *R*^2^ values < 0.99. Losses during extraction or RT-qPCR were not used to calculate values for bacterial or viral GC and therefore reported values represent uncorrected values.

### Estimate of bacterial non-culturability

Estimates of non-culturability were determined by measuring the fold difference between total gene copies (GC/100 ml water or GC/100 g sediment) as measured by qPCR and culturable bacteria (CFU/100 g sediment) measured by microbiological plate count technique.

### Sediment particle size analysis

Sediment grain size was determined by laser diffraction after 1 min sonication to separate particles, using a particle size analyser (Malvern Hydro 2000, UK) in conjunction with the Mastersizer 2000 software. Three replicate sediment samples from each site were individually homogenized. Approximately 1 g of sediment was added to the particle size analyser and 3 independent size determinations were made, for which the average grain size and sedimentary rock classification was determined using insert grain size software (Gradistat 4.0).

### Determination of sediment organic matter content, elemental composition and density

The loss-on-ignition method (LOI) was used to determine organic matter content of sediment samples. Approximately 20 g of fresh sediment from each sample was placed in a pre-weighed crucible and dried at 95°C for 24 h. Approximately 5 g of the resultant dried sediment was weighed, transferred to another crucible and placed into a muffle furnace at 550°C for 6 h. Organic matter content was calculated as the difference between the weight of the dry sediment and weight of the residue post-combustion. Moisture content per g of fresh sediment was determined by calculating the percentage difference between wet weight and dry weight after 24 h at 95°C (APHA, 2012). The elemental composition of the sediment was determined on finely ground duplicate samples using the S2 Picofix total reflection X-ray fluorescence (TXRF) spectrometer (Bruker Ltd., UK). Particle density was determined by the pycnometer method and bulk density was calculated by the weight of a known volume of sediment subsequently. Porosity was estimated from the bulk density and particle density measurements according to Flint and Flint (2002).

### *In situ* physicochemical measurements of estuarine sample sites

Temperature and depth measurements were recorded *in situ* using vessel-mounted probes. Electrical conductivity and pH was assessed post-sampling using standard electrodes and salinity was estimated based on temperature corrected conductivity values from 8th degree polynomial fit to standards. Depth could not be determined at the Ribble estuary due to shallow water across the mudflats which prevented use of the depth probe.

### Statistical analysis

Using SPSS v22 (IBM Corp., Armonk, NY), correlations were performed using the data for each site to determine the relationships between cultured bacterial abundance, qPCR gene copies in sediments and water with physicochemical parameters. Data were transformed (log_10_N+1) and a two-tailed bivariate Spearman's rank correlation was performed. Statistical analyses were performed using Primer v7 with PERMANOVA+ add on (Clarke and Gorley, 2015) to explore relationships between bacterial community changes. For culture-dependent and molecular methods, results below detection limit were assumed as half the detection limit to allow comparative assessment using previously accepted methods to analyse sites with low microbial contamination (Henry et al., 2016b). Dummy values were added (e.g., +1) to all culturable bacterial counts and qPCR gene copies to remove missing abundance values and permit calculation of Bray-Curtis similarity coefficient. Data were log_10_ transformed to downweight the most abundant genera, next dissimilarities were calculated with the S17 Bray-Curtis similarity coefficient. Environmental variables were normalized to a common scale (by subtracting the mean and dividing through by the standard deviation). Euclidean distances calculated between physicochemical variables was used to quantify resemblance between sites. A principal coordinate ordination analysis was performed by plotting the inter-point dissimilarities values for each factor (site and sampling events), the variation in community composition was plotted as the first two axes (preserving actual dissimilarities) (Gower, 1966). A correlation was performed between environmental variables and each community coordinate. Correlations with each component were deemed significant if *R*^2^ > 0.5 and a vector biplot was overlaid to visualize the strength of the correlation. A parametric Welch's *t*-test was performed to test significance of differences between key taxa and physicochemical variables for each transect/sampling events (season), with significance *p* < 0.05 deemed significant. A Games Howell *Post-Hoc* test was undertaken to assess site/sampling event differences. The abundance of *E. coli* and *Enterococcus* spp. was transformed (if required) and plotted as a heat map. Bathing water standards based on 2006/7/EC were overlaid on the heatmap. Bathing Water Standards were used to aid interpretation of the bacterial datasets (quantified by qPCR and plate counts) and not to assess compliance which is based on specified statistics for a defined period of time.

### Distance based linear modeling

Multivariate distant based linear model (DistLM) were constructed to assess the effect of different physicochemical features on the total abundance (qPCR) and culturability of key bacteria. Predictor variables were often not independent and partly correlated with each other. Therefore, BEST analysis was used to distinguish most suitable water or sediment characteristics for predictive analysis. A Durbin Watson test was undertaken and values ~2 suggested independence of observation. Correlations between predictor variables was assessed and variables with autocorrelations *R* > 0.8 were excluded from the analysis, by taking the most suitable variable forward for the model construction. Tolerance values were calculated from the data and samples with a tolerance value >0.1 were excluded due to potential multi-collinearity. Finally, a resemblance matrix based on Bray-Curtis similarity index was compiled using a step-wise forward procedure with 9999 permutations (PERMANOVA+ for PRIMER; Anderson et al., 2008). A Distance Based Redundancy Analysis (dbRDA) was undertaken on significant predictor variables and visualized by plotting dbRDAs coordinate scores in two-dimensional space.

## Results

### Environmental variables

The water temperature in the Ribble in July, was 10°C warmer in (sites 1–2) and 5°C warmer (sites 3–8) than Conwy transect averages (*p* < 0.01) in June, probably due to seasonal warming between sample dates undertaken for each location, poor mixing and higher wastewater discharges compared to the Conwy. However, in April the Ribble water temperature was also ~3°C higher than the Conwy (*p* = 0.05). During other sampling events, the water temperature was similar between locations (Figure 2A). The salinity profile of the Conwy ranged from estuarine to fully saline conditions (15–39 PSU) from transect 1 to transect 5 (Figure 2B). The salinity in the Ribble was typically lower ranging from 11 to 22 PSU (Sites 3–8) respectively (Figure 2B). Turbidity was greater in the Ribble sites 1–2 than other sites Ribble or sites in the Conwy (Figure 2D). Sites 3–8 in the Ribble had a turbidity of 85 (range 52–167) NTU in June 2015 possibly due to resuspension events caused by adverse weather on this sample event (Figure 2D). The pH in the Ribble sites 1–2 was ~7.7 which was consistently lower than the other sites in the Ribble or Conwy (Figure 2C). Sediment porosity was similar between transects and did not differ between the Ribble and Conwy (Figure 2F). Sediment sampled from the mudflats in the Conwy or the Ribble had a greater clay content (~40%) compared to other sites < 20% (*p* < 0.001) (Figure 2G). Sediment organic matter content did not display seasonal trends (Figure 2H) and was probably dependent on local hydrodynamics which govern sediment transport and deposition and organic matter accumulation.

### Bacterial/pathogen loading in water

**Figure 3:**
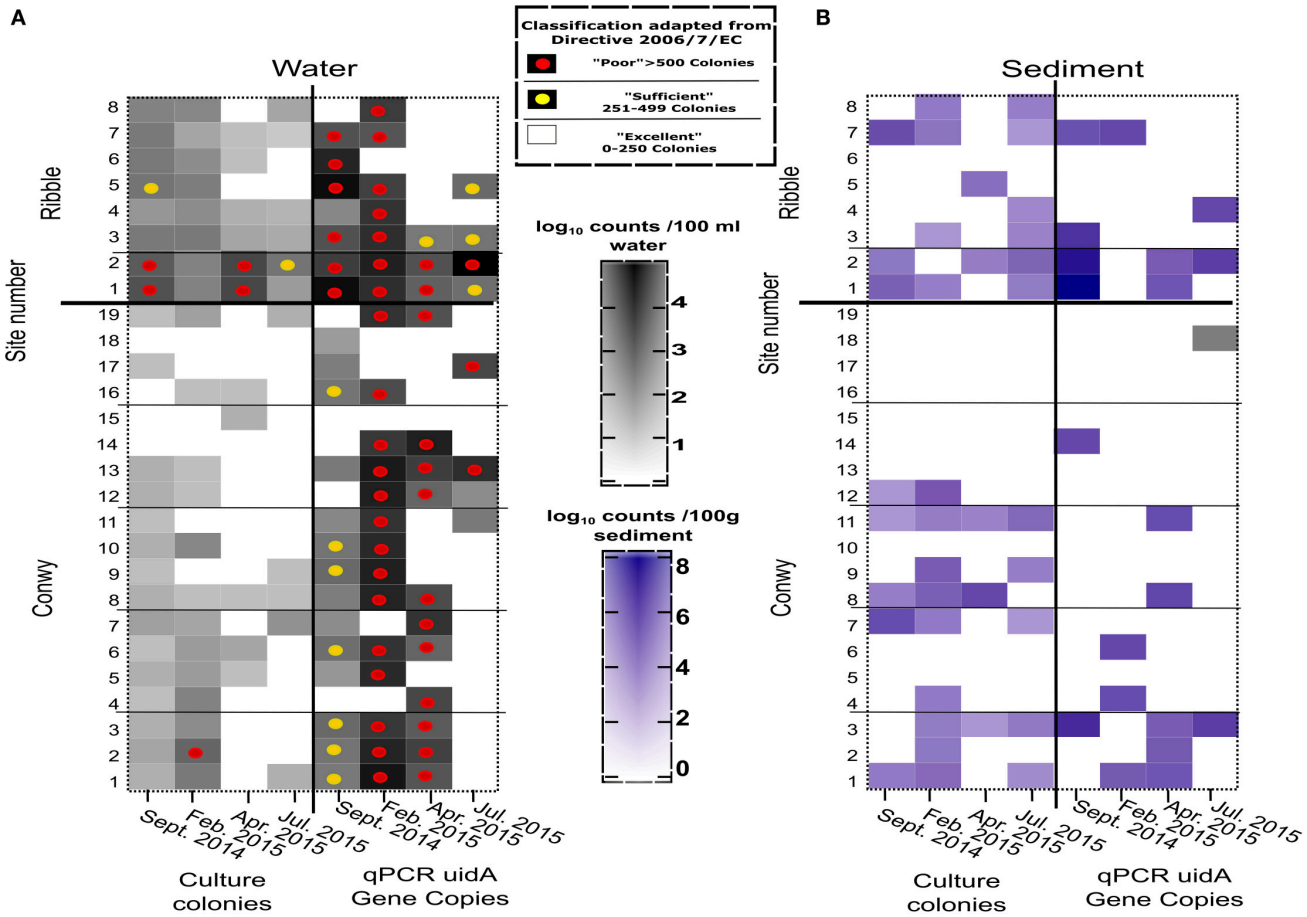
*E. coli* abundance in the Conwy estuary (sites 1–19) and the Ribble estuary (sites 1–8) for sampling events **(A)** Water (CFU/100 ml or GC/100 ml), **(B)** Sediment (CFU/100 g or GC/100 g wet weight). Sample sites compliance indicated by superimposed circles for Water Framework Directive 2015 2006/7/EC for Water (see box). Samples without circle indicates better than “excellent” water quality for Coastal and Transitional Waters.

**Figure 4:**
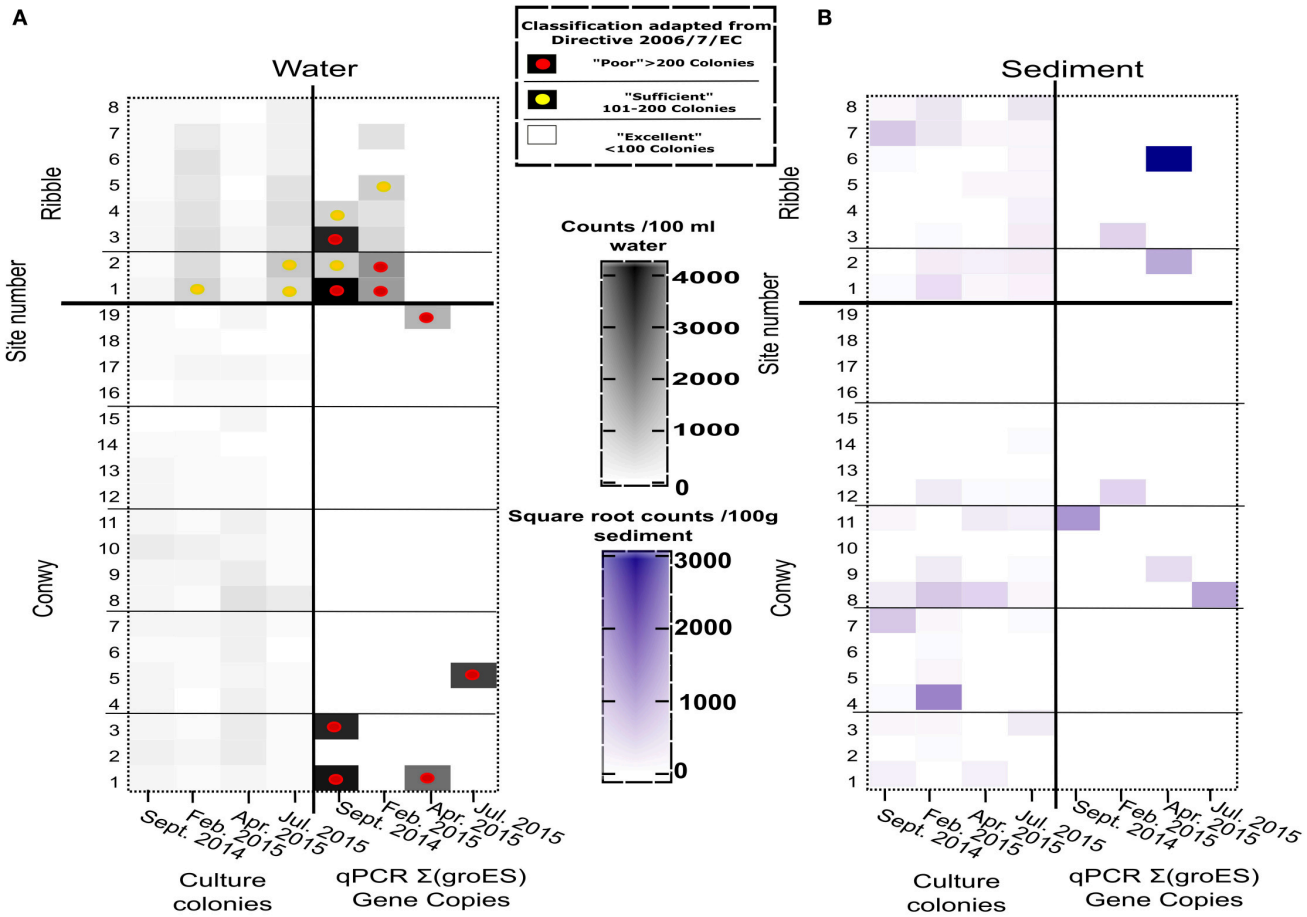
**(A)** Intestinal enterococci abundance in the Conwy Estury (sites 1–19) and the River Ribble (sites 1–8) for sampling events (Water (CFU/100 ml or GC/100 ml), **(B)** Sediment (CFU/100 or GC/100 g wet weight). Sample sites compliance indicated by superimposed circles for Framework Directive 2015 2006/7/EC for water (see box). Samples without circle indicates better than “Excellent” water quality for Coastal and Transitional Waters. qPCR copies represent sum of Enterococcus *faecium and E. faecalis*.

The concentrations of *E. coli* varied considerably with site and sampling events (Figure 3A). The greatest *E. coli* abundance in the Conwy was 274 (range < 1–522) CFU/100 ml at transect 1 (sites 1–3), in February 2015. The bathing water quality classifications for *E. coli* and intestinal enterococci are presented as a simplified version of the bathing water quality thresholds (Figures 3, 4). This provides a representation of the FIO abundance and the “potential” water quality status between each site/sampling event and relative difference between plate count and qPCR methods for quantification, this approach is to aid interpretation of the data and not to assess compliance. qPCR based methods are not being utilized to assess compliance to water quality standards in the EU or USA. However, this could change with requirements for culture-independent requirements for bacterial or viral quantification (Hassard et al., 2017). There was a single “poor” classification at site 2 (transect 1) in February 2015 (Figures 1C, 3A). In contrast, the greatest abundance in the Ribble was at sites 1 and 2 in April 2015, with an average *E. coli* abundance of 2,367 CFU/100 ml in water. Sites 3–8 in the Ribble had <50 CFU/100 ml in both April and July suggesting low levels of fecal contamination.

The Conwy estuary had excellent water quality for intestinal enterococci abundances typically <100 CFU/100 ml (Figure 4A). Intestinal enterococci abundance was lowest in June 2015 at 2 CFU/100 ml and greatest in April 2015 at 12 CFU/100 ml (based on average values). The number of *E. coli* detected in water using qPCR ranged from below limit of detection (<1 GC/100 ml) to a maximum of 7.2 × 10^5^gene copies (GC)/100 ml (Figure 3A) in Ribble site 2 in July 2015.

The Ribble sites 1 and 2 had significantly higher *E. coli* CFU counts in the water column compared to other sites in the Ribble or the Conwy. There were four sites which had >500 *E. coli* CFU/100 ml, suggesting poor water quality. Bacterial water quality was highly location-specific with the Ribble having 12.5% (4/32) of sites classified as “poor” compared to 1.3% (1/76) of samples taken in the Conwy based only on culture data (Figure 3A). However, qPCR analysis revealed the potential for “poor” water quality was 50% (16/32) of samples taken in the Ribble, which was less in the Conwy at 35% (27/76) of samples (Figure 3A) (Welch's *t*-test on pooled sites *p* < 0.001). All sites in the Conwy were had culturable intestinal enterococci (<100 CFU/100 ml). In contrast, 9% (3/32) of samples were deemed “sufficient” for intestinal enterococci in the Ribble. Potential for poor water quality for *Enterococcus faecalis, E. faecium* was 5.2% (4/76) in the Conwy and 12.5% (4/32) in the Ribble based on qPCR data on two species (Figure 4A). Low levels of *Salmonella* spp. (typically 10-111 GC/100 ml) were detected in Conwy waters in 63% of sites in April (12/19) and 21% of sites in May (4/19) possibly due to the presence of migratory birds or wastewater discharge (Table S3). We found low levels (<10 GC/100 ml) of *Campylobacter jeujuni* at the Conwy site 19 in water in April 2015. We did not detect pathogenic *Shigella* spp. or the pathogenic hepatitis A virus or hepatitis E virus. In contrast, we detected <10 GC/100 ml of norovirus GII in the water at sites 1 and 2 in the Ribble, suggesting low level contamination near the effluent discharge point of Preston Clifton Marsh WwTW. In this study, qPCR analysis may also have amplified the target genes of DNA derived from exogenous sources and dead cells.

The potential effect of physicochemical controls on bacterial abundance (Table S2) and culturability (Table S1) to season (represented by 4 sampling events) and location (2 estuaries) was tested by multiple regression (DistLM), while additionally the results were visualized by dBRDA (Figure S1). When testing across season and location all studied variables contributed in a significant way to the variability in bacterial abundance (*p* < 0.001) (Table 1) with salinity and pH accounting for 16 and 12% of the variability in bacterial abundance. When separating by season, it became clear that temperature accounted for 60%, of the variability in bacterial abundance in autumn (*p* < 0.001). In winter, salinity accounted for a further 15% (*p* < 0.001) of the variance. This was at the expense of temperature which accounted for 23% (*p* < 0.001) of the variability in abundance. These factors account for a substantial and significant component of the *E. coli* abundance and change seasonally (Figures 2A,B, 3A). In spring, bacterial abundance was governed by water turbidity (33%) suggesting a possible link between abundance and organic matter content or suspended particulate matter (Table 1).

**Table 1:** Distance Based Linear Models (DistLM) sequential tests.

	***R*^2^**	**SS**	**Pseudo-F**	***p***	**Proportion of variation (%)**
**RESPONSE VARIABLE: log_10_(BACTERIAL COUNT)/100 ml WATER**					
**September and October**					
Temperature	0.59	5,750	36.6	**<0.001**	59.5
Turbidity	0.66	644	4.7	**0.002**	6.7
**February**					
Temperature	0.23	1,516	7.5	**<0.001**	23.1
Salinity	0.37	972	5.7	**<0.001**	14.8
pH	0.48	690	4.7	**0.008**	10.5
**April**					
Turbidity	0.33	4,303	12.5	**<0.001**	33.3
Salinity	0.43	1,241	4.1	**0.016**	9.6
Depth	0.48	687	2.4	0.086	5.3
**June and July**					
Turbidity	0.48	7,658	23.5	**<0.001**	48.4
Temperature	0.56	1,200	4.2	**<0.001**	7.6
pH	0.62	879	3.3	**0.022**	5.6
**RESPONSE VARIABLE: log_10_(BACTERIAL COUNT)/100 g SEDIMENT**					
**September and October**					
Mean grain size	0.14	4,030	3.3	**0.008**	13.8
Clay (%)	0.21	2,068	1.8	0.182	7.1
Porosity (%)	0.28	1,970	1.8	0.193	6.7
Zn	0.36	2,575	2.5	0.084	8.8
S	0.43	1,996	2.0	0.146	6.8
Al	0.55	3,302	4.0	**0.023**	11.3
**February**					
Clay (%)	0.17	4,853	4.3	**0.007**	16.9
Ni	0.26	2,747	2.6	0.066	9.6
Cu	0.38	3,298	3.5	**0.036**	11.5
Mn	0.45	2,005	2.3	0.082	6.9
Zn	0.51	1,847	2.2	0.099	6.4
Mean grain size	0.58	1,791	2.4	0.071	6.2
**April**					
Ca	0.24	9,212	6.5	**<0.001**	23.7
Nominal density	0.39	5,788	4.8	**0.007**	14.8
K	0.59	4,100	4.6	**0.012**	10.5
Fe	0.68	3,561	4.9	**0.013**	9.2
Organic matter content (%)	0.75	2,731	4.5	**0.020**	7.0
**June and July**					
Mean grain size	0.19	3,927	4.9	**0.005**	18.8
Porosity (%)	0.29	2,099	2.8	0.067	10
Zn	0.41	2,525	3.9	**0.026**	12
Clay (%)	0.55	2,983	5.7	**0.004**	14.2
K	0.69	1,370	3.2	**0.042**	6.6
Al	0.76	1,300	3.6	**0.021**	6.2

*Response variables are log_10_ bacterial abundance in water (CFU/100 ml and GC/100 ml) and sediment (CFU/100 g and GC/100 g) for GC (Escherichia coli, Enterococcus faecalis, E. faecium, Vibrio spp., Campylobacter jeujuni, Shigella spp., Salmonella spp.) and CFU (Vibrio presumptive, E. coli, coliforms and Enterococcus). Predictor variables for water include temperature, salinity, pH and turbidity. Predictor variables for sediment include: mean grain size, clay content, porosity, Zn, S, Al, Cu, Ni, Mn, K, Fe, depth and organic matter content. p-values p < 0.05 are deemed significant and are in bold for sequential test. Factors are included if they represent >5% of the variation*.

### Bacterial/pathogen loadings in sediment

Pathogenic bacterial species were not detected in the sediments of the Conwy or Ribble estuaries by qPCR (Table S4), suggesting true absence or methodological limitation (e.g., PCR inhibition). Sediment culturable *E. coli* loadings varied considerably with site and sampling event (Figure 3B) (*p* < 0.05). The abundance of culturable *E. coli* in Ribble sediments was approximately double the abundance in Conwy sediments (4.8 and 4.6 log_10_ CFU/100 g) respectively (pooled event data for each location excluding sites below detection limit). In February 2015, the numbers of *E. coli* in sediments showed some differences within locations ranging from below detection limit to 3.9 log_10_ CFU/100 g in the Ribble sites 1–2 to a maximum of 5.1 (5–5.2) log_10_ CFU/100 g in the Conwy transect 3 (Figure 2B, *p* = 0.034). Culturable *E. coli* was greatest at 8.2 log_10_ CFU/100 g sediment at Ribble site 1 and was not detectable (<100 CFU/100 g) at sites 13–19 in Conwy and sites 5 and 6 in the Ribble irrespective of season (*p* = 0.035). This trend was reflected in the intestinal enterococcus data, but not with the *Vibrio* data which ranged from 4.9 to 5.5 log_10_ CFU/100 g sediment (Table S4), highlighting the marine preference of this group and a seasonal temperature dependency (Figure 2A). On a transect basis, the minimum *Vibrio* abundance was 2.7 (range 2–3.2) log_10_ CFU/100 g sediment in Conwy transect 4 and a maximum of 5.2 (range 2–5.8) log_10_ CFU/100 g sediment in Conwy transect 2 in September 2014. In September 2014, *E. coli* could not be detected in sediments at transect 2, 3, and 5 in the Conwy by qPCR. *E. coli* detection ranged from a minimum of 1.9 (range 2–5.8) log_10_ CFU/100 g sediment in Conwy transect 4 compared to a maximum of 7.9 (range 7–8.2) log_10_ CFU/100 g sediment in Ribble sites 1–2 (Figure 3B) (*p* = 0.05). Conwy transect 1 and Ribble sites 1–2 had ~5.3 log_10_ CFU/100 g sediment, suggesting elevated levels at these sites (*p* < 0.001) (Table S4) which was similar to the trends observed in water.

The maximum number of culturable *Enterococcus* detected in sediments was in 6.1 log_10_ CFU/100 g in transect 2 in February 2015. Lower qPCR *Enterococcus* abundance was detected with abundances of 5.2 (range 2–5.8) log_10_ GC/100 g in Conwy transect 3, with much lower detection of *Enterococcus* observed in other transects in this sampling event (Table S4, Figure 4B) (*p* = 0.046). Sediments which were positive for FIOs or enteric pathogens typically had a greater relative abundance of FIOs or enteric pathogens than the overlying water column. Sediment resuspension could therefore influence water quality and compliance at a local level. When testing across season and location, the grain size (10%), salinity (5%), K concentration (3%), and bulk density (3%) together explained a significant and substantial component of the variation in bacterial abundance (*p* < 0.03) (Table S5), although in isolation each variable typically accounted for <10% each to the bacterial abundance, highlighting the heterogeneous nature of the sediment matrix and therefore the difficulty in modeling sediments across large spatial scales.

### Impact of seasonal trend on bacterial abundance and culturability in water

When testing across season and location, it was found that temperature and salinity accounted for 14% (*p* = 0.001) and 10% (*p* = 0.03) respectively of the variation in bacterial non-culturability in the water (Table S5). The non-culturability index for *Vibrio* spp was 4,661 (range 54–66,220) qPCR/CFU counts on average, in the Ribble. This is higher than 996 (range 1–27,050) qPCR/CFU counts in the Conwy estuary (*p* < 0.001) (**Figure 6A**) suggesting that the bacterial groups studied here were more likely to be non-culturable in the Ribble. In contrast, the non-culturability index for *E. coli* in water was 1,344 (range 1–20,802) qPCR/CFU counts on average in the Conwy compared to 28 (range 1–257) qPCR/CFU counts in the Ribble water (*p* < 0.05) (**Figure 6B**). Elevated salinities and low water temperatures contribute to a high non-culturability index of *E. coli* in the Conwy (**Figures 6A,B**, Figure S1B). Low temperature in winter was the largest contributor to the low culturability of *E. coli* and *Vibrio* (32%, *p* < 0.001) (Figures 3A, **6**, Table 2). Non-culturability index could not be calculated for *Enterococcus* species as the culture media utilized has low selectivity for intestinal enterococcus and qPCR primers were specific for the principal enteric species *E. faecalis* and *E. faecium* resulting in lower qPCR counts than CFU (values <1 for non-culturability).

**Table 2:** DistLM sequential tests.

	***R*^2^**	**SS**	**Pseudo-F**	***p***	**Proportion of variation (%)**
**RESPONSE VARIABLE: WATER BACTERIAL FOLD CULTURABILITY (qPCR/CFU)**					
**September and October**					
Turbidity	0.17	4,821	5.3	**0.012**	17.4
pH	0.23	1,532	1.7	0.187	5.5
**February**					
Temperature	0.32	11,741	11.7	**<0.001**	31.9
Salinity	0.58	1,790	2.5	0.104	4.9
**April**					
Turbidity	0.22	2,001	1.5	0.251	4.8
**June and July**					
Turbidity	0.14	3,221	4.2	**0.036**	14.4
Temperature	0.25	2,267	3.2	**0.044**	10.2
**September and October**					
Sand + silt (%)	0.29	2,985	2.5	0.121	8.9
Clay (%)	0.35	1,962	1.7	0.194	5.9
Al	0.47	3,196	3.1	0.079	9.6
S	0.58	3,653	4.2	**0.039**	10.9
Mn	0.62	1,342	1.6	0.215	4.0
**February**					
Organic matter content (%)	0.15	2,086	3.7	**0.050**	14.9
Porosity (%)	0.35	2,847	6.3	**0.021**	20.3
Ca	0.50	2,114	5.8	**0.020**	15.1
S	0.65	2,051	7.6	**0.007**	14.7
**April**					
Zn	0.24	5,649	6.6	**0.013**	23.8
S	0.43	4,460	6.5	**0.017**	18.8
Porosity (%)	0.60	4,231	8.6	**0.007**	17.8
Cl	0.67	1,543	3.5	0.063	6.5
K	0.73	1,366	3.6	0.066	5.8
Cu	0.82	1,388	4.6	**0.038**	5.8
**June and July**					
P	0.18	4,714	4.7	**0.035**	18.2
Mean grain size	0.29	2,749	2.9	0.086	10.6
Clay (%)	0.44	4,034	5.3	**0.023**	15.6
Cl	0.53	2,227	3.3	0.068	8.6
Br	0.69	4,091	8.7	**0.004**	15.8
Cu	0.78	2,381	6.8	**0.012**	9.2

*Response variables are culturability fold difference between bacterial target counts log_10_(qPCR/CFU) (groups included Escherichia coli, Enterococcus, Vibrio) in water and sediment. Predictor variables for water include temperature, salinity, pH and turbidity. Predictors variables for sediment include: mean grain size, clay content, non-clay content, porosity, Ba, Al, S, Mn, Ca, P, Cu, and depth. p-values p < 0.05 are deemed significant and are in bold for sequential test. Factors are included if they represent >5% of the variation*.

### Sediment elemental composition consistency within estuaries

PERMANOVA analysis revealed the total sediment elemental composition (SEC) did not vary with sampling event (Table S5) based on pooled site data (*p* > 0.05). However, the SEC (the sediment elements included in analysis are detailed in Table S6) in the Conwy was substantially different from the Ribble (*p* = 0.009), notably in Ca concentration, where, the Ribble had 11.6 ± 0.8 g/kg and the Conwy had 6.6 ± 1.1 g/kg (*p* = 0.003) (Table S6). In addition, Zn concentration was 66 ± 7.7 mg/kg in the Conwy and 29 ± 4.3 mg/kg (*p* = 0.001) in the Ribble. We speculate that historic mining activity could have led to heavy metal contamination in the Conwy catchment. BEST analysis (utilizing BIOENV and Spearman's rank correlation) on individual samples revealed that of the 27 elements studied; Al, Br, Ca, Cu, Fe, K, P, S, and Zn were the best potential predictors of sediment-bound bacteria. Therefore, these elements were taken forward for modeling purposes and average concentrations are presented in Table S6.

### Relationship between bacterial abundance, non-culturability and physicochemical parameters in sediments

The sediment compartments which correlated with *E. coli* and *Enterococcus* spp. were (in order from most significant to least significant correlation): very fine sand>clay>silt (Table 3). In contrast, coliforms positively correlated with very fine and fine sand, and negatively correlated with medium sand and coarse sand. *Vibrio* spp. correlated to a lesser degree to very fine sand>clay>fine sand>silt. All groups negatively correlated with medium sand (*p* < 0.01; Table 3). Cohesive sediments demonstrated a positive association with the bacteria studied, whereas fine sediments (63–125 μm) and coarse sediments (>125 μm) had a negative correlation with bacteria. When separating by season, the S concentration (>11%, *p* = 0.039) was an important predictor of non-culturability in autumn, whilst in winter the sediment organic matter content, porosity, Ca and S concentration >15% per variable (*p* < 0.05) were important. In spring, sediment Zn and S concentration and porosity (>17% per variable, *p* < 0.05) explained bacterial abundance. In summer, P concentration, clay content, Br and Cu each explained >15% (*p* < 0.05) of the variation in bacterial abundance in sediments (Table 2).

**Table 3:** Spearman's rank correlations of different culturable bacterial groups with sediment particle size fractions.

**Target organism**	**Particle size fraction**						
	**Clay**	**Silt**	**V. fine sand**	**Fine sand**	**Medium sand**	**Coarse sand**	**V. coarse sand**
*E. coli*	**0.33**	**0.25**	**0.32**	−0.06^*^	−**0.28**	−0.19^*^	−0.20
Coliforms	0.18^*^	0.10^*^	**0.45**	0.24^*^	−**0.30**	−0.22^*^	−0.14
*Vibrio* spp.	0.27^*^	0.21^*^	0.43^*^	0.24^*^	−**0.32**	−0.24^*^	−0.07
*Enterococcus* spp.	**0.44**	**0.42**	**0.48**	−0.11^*^	−0.26	−0.13^*^	−0.09
Heterotrophs (Marine)	0.25^*^	**0.14**	**0.37**	−0.12	−0.25	0.06	0.07
Heterotrophs (R2A)	0.02	−0.05	0.19^*^	0.02	0.02	0.00	0.06

*Bold numbers indicate correlation is significant at the P < 0.01 level (2 tailed)*.

* *Numbers indicate correlation is significant at the P < 0.05 level (2 tailed)*.

*Vibrio* spp. non-culturability in sediments was similar with season at ~2 log_10_ qPCR/CFU (**Figure 6C**). This suggests that 100-fold more genome copies were present in both Conwy and Ribble than culturable bacteria and that season did not influence this. In the water column, the non-culturability was higher at 2.2–3.8 log_10_ qPCR/CFU. *E. coli* non-culturability in sediments was typically 1.5–2.2 log_10_ qPCR/CFU for the Ribble and Conwy irrespective of season (**Figure 6D**). An exception to this occurred in April 2015 where both the Conwy and the Ribble had ~5.5 log_10_ qPCR/CFU ratios. Levels of Zn were 2.4-fold higher in the Conwy than in the Ribble which may have influenced culturability of sediment-bound bacteria. The sediment Zn concentration significantly influenced the culturability of bacteria (19%, *p* = 0.013) (Table 2).

## Discussion

### Impact of spatial patterns in physicochemistry on the detection of fecal contamination coastal and estuarine waters

In this study water turbidity was an important determinant of the abundance (Table 1) and culturability (Table 2) of *Escherichia coli, Enterococcus faecalis, E. faecium*, and *Vibrio* spp. in spring and summer, respectively. This could be due to the stabilizing effect of suspended particulate matter on bacteria (Gin and Goh, 2013; Perkins et al., 2014; Prasad et al., 2015; Hassard et al., 2016), and the enhanced attachment of gammaproteobacteria to particles (Liu et al., 2016). Particulate association may mitigate the harmful effects of UV, which could exert a strong effect on bacterial viability in shallow coastal and estuarine regions (Kay et al., 2005). In a different study, turbidity levels >200 NTU was found to completely inhibit the UV-induced decay of intestinal enterococcus (Kay et al., 2005). Peaks in turbidity of 35 and 28 NTU in Sept./Oct. 2014 and April 2015 contributed to elevated bacterial abundance particularly in the Ribble sites 1 and 2. Therefore, when assessing risk factors, water turbidity or suspended particulate matter could represent a suitable tool for predicting water quality and undertaking microbial risk assessment. Regulation and management practices could be adapted to accommodate spikes in water quality/turbidity by temporarily closing bathing waters or shellfish harvesting areas during periods of disturbance (Shah et al., 2007).

**Figure 5:**
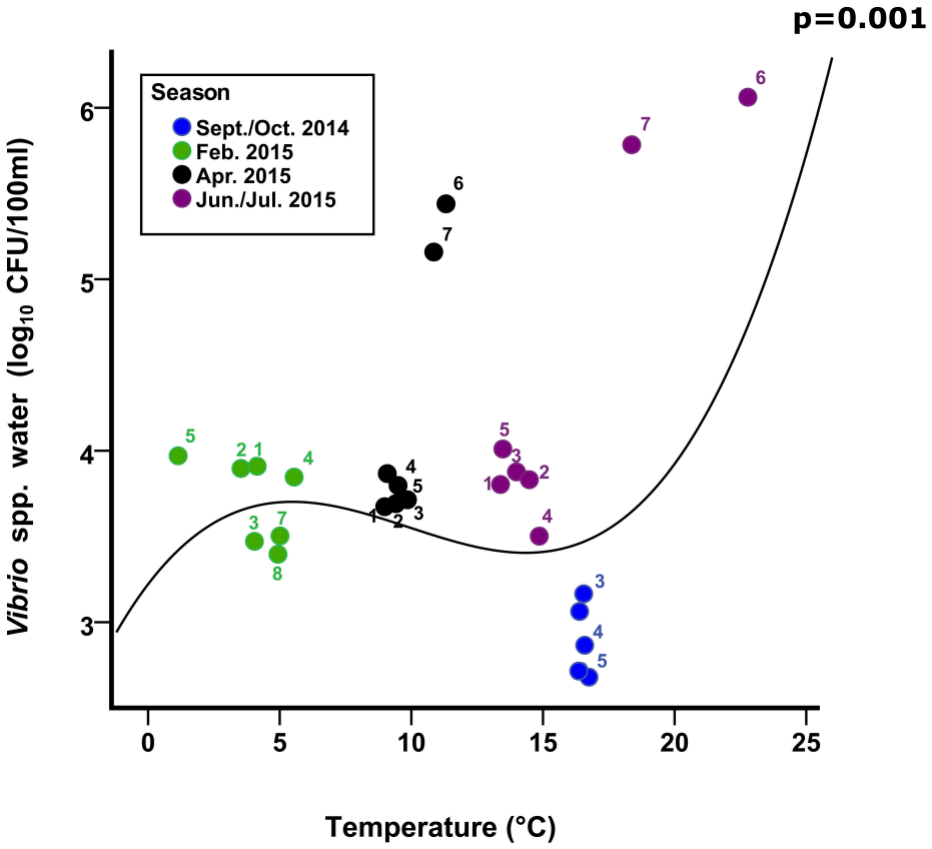
Culturable *Vibrio*. Numbers represent the mean abundance and mean temperature (corresponding SD are presented in Figure 4) for each Conwy transect 1–5 and Ribble sites 1–2 (6) and Ribble sites 3–8 (7). Line represents cubic regression of *Vibrio* spp. in Conwy and Ribble water with water temperature, *p*-value highlights significance of fit to data.

In this study, *E. coli* and *Vibrio* populations in the water column responded differently. For example, a reduction in *Vibrio* culturability occurring in the Ribble water column in winter (Figure 5), probably due to low temperature and low salinity, which are important stressors, reducing the culturability of *Vibrio* (Table 2). In contrast, *E. coli* abundance increased with elevated turbidity (17%) and the lower temperatures (31%) in autumn and winter respectively. This study does not consider run-off effects or local dredging/boat action on turbidity, only that turbidity is a useful predictor of bacterial loading within this study.

We found water temperature represented a substantial and significant component of culturable bacterial abundance (negative for *E. coli*, positive for *Vibrio*) which agrees with trials undertaken in controlled conditions (Ayrapetyan et al., 2014; Abia et al., 2016), and that the trend in this study was similar between the two very different estuaries (Figure 1). This is remarkable considering strong gradients in other environmental variables, particularly salinity in the Conwy estuary (Figure 2B). The negative correlation between temperature and *E. coli* abundance suggests better survival in winter or that the magnitude of the sources is greater (Kay et al., 2005; Neave et al., 2014; Campos et al., 2016), however, the high non-culturability in the Ribble sediment in winter suggests local adaptation to physicochemical conditions or enhanced persistence of environmental DNA. Sinigalliano et al. (2010) found a negative correlation between water temperature, respiratory infections and abundance of culturable intestinal enterococcus, suggesting pathogenicity and culturability are linked, and subject to physicochemical factors (Lodder et al., 2015).

In this study, the greatest culturable abundance of *Vibrio* occurred in June/July 2015, despite greater concentrations in overall *Vibrio* spp. genome copies in September/October 2014. This suggested that in autumn another factor limited culturability, possibly organics/nutrient concentrations (Thorn et al., 2011; Quilliam et al., 2014; Shelton et al., 2014). Declining FIO abundance, typically from river to estuary mouth, was linked to increased salinity or dilution effects (Anderson et al., 1979). The Ribble sites 1 and 2 had a greater culturable and qPCR abundance in water and sediments compared to most other sites in the Ribble and Conwy estuaries, this is possibly due to a large WwTW (PE > 100,000) downstream of site 1 and upstream of site 2. A recent study showed that tidally-driven hydrodynamics determined the dispersal and concentrations of bacterial indicator/pathogen concentrations near a wastewater outfall plume (Winterbourn et al., 2016). Soluble and particulate tracer studies (e.g., Drummond et al., 2014) could therefore yield valuable information on the transport, retention dynamics and impact of sediments on accessibility and infectivity of bacterial indicators and pathogens (Moore et al., 2013), within complex estuarine systems such as the Conwy and the Ribble.

### Effect of sediment composition governing spatial patterns of sediment associated bacterial populations

In this study, 32% of sites in the Conwy and 38% of sites in the Ribble had lower relative bacterial abundance in sediment than found in the overlying water column based on average abundance for *E. coli*. Previous studies (e.g., Vignaroli et al., 2013) have suggested this could be due to sunlight inactivation or resuspension reducing the bed bound bacterial load (and increasing the water bacterial load) prior to monitoring. In this study, most of the sites where lower sediment bacterial abundance occurred were associated with coarser sediments (grain size >250 μm) e.g., Conwy sites 13–19 (Figure 2E). This suggests that low sediment bacterial abundance could be linked to poor retention of enteric bacteria and viruses in coarse sediments compared to fine sediments, or poor recovery of bacteria/viruses from these sediments (Howell et al., 1996; Jamieson et al., 2005). Bacterial accumulation occurs within the unstable, top 3 cm of the sediment within estuarine environments (Drummond et al., 2014). Mounting evidence also suggests that epibenthic flora/fauna/debris could be important reservoirs of fecal bacteria/viruses (Quilliam et al., 2014).

In estuaries, free floating bacteria and viruses may be exposed to large gradients in salinity and over small spatial scales this can result in flocculation and deposition of fine particulates with bound bacteria and viruses (Malham et al., 2014). Furthermore, elevated salt levels can increase the attraction of bacteria/viruses to and adhesion with sediment particles (Cai et al., 2013). Sediment types also influences the viability and culturability of *E. coli*. We have demonstrated the positive association of different bacterial groups to cohesive particles (<63 μm) such as clays (Table 3) (Jamieson et al., 2005; Pachepsky and Shelton, 2011). However, different types of clays with a net positive charge (e.g., goethite) were found to have superior binding affinity to *E. coli* O157:H7 compared to clays which had a net negative charge (e.g., kaolinite and montmorillonite) (Cai et al., 2013). Elevated levels of elemental Ca in the Ribble governed a small but significant component of the sediment bound bacteria (Table 1), potentially due to attraction of bacteria and viruses with a net negative charge. In contrast, we found greater levels of elemental Zn in the Conwy which governed a small amount of sediment bacterial abundance. Zn is cytotoxic to prokaryotes at concentrations >6.6 mg/kg. The potency of Zn as an antimicrobial is dose dependent (Bitton et al., 1972), and in the Ribble and Conwy values were ~6 × and ~10 × greater respectively than this threshold which could explain the lower abundances of bacteria in the sediments in the Conwy compared to the Ribble (Tables 2, 3), highlighting a possible conditioning effect of Zn on bacterial communities (Pasquet et al., 2014). Strong interaction with positively charged surfaces (e.g., from other cations not included in the models here) can inhibit bacterial growth and reduce culturability/viability (Gottenbos et al., 2001; Van Der Mei et al., 2008).

The positive link between sediment organic matter content and FIOs is well established (Halliday and Gast, 2011; Pachepsky and Shelton, 2011; Perkins et al., 2014). In this study, we found that organic matter content could account for 7% of the bacterial variability in spring 2015. This highlights the complexity of the sediment matrix and that bacterial indicators within sediment may not adequately represent the extent of fecal pollution in the environment.

### Should we rely on culture dependent methods to indicate fecal pollution?

**Figure 6:**
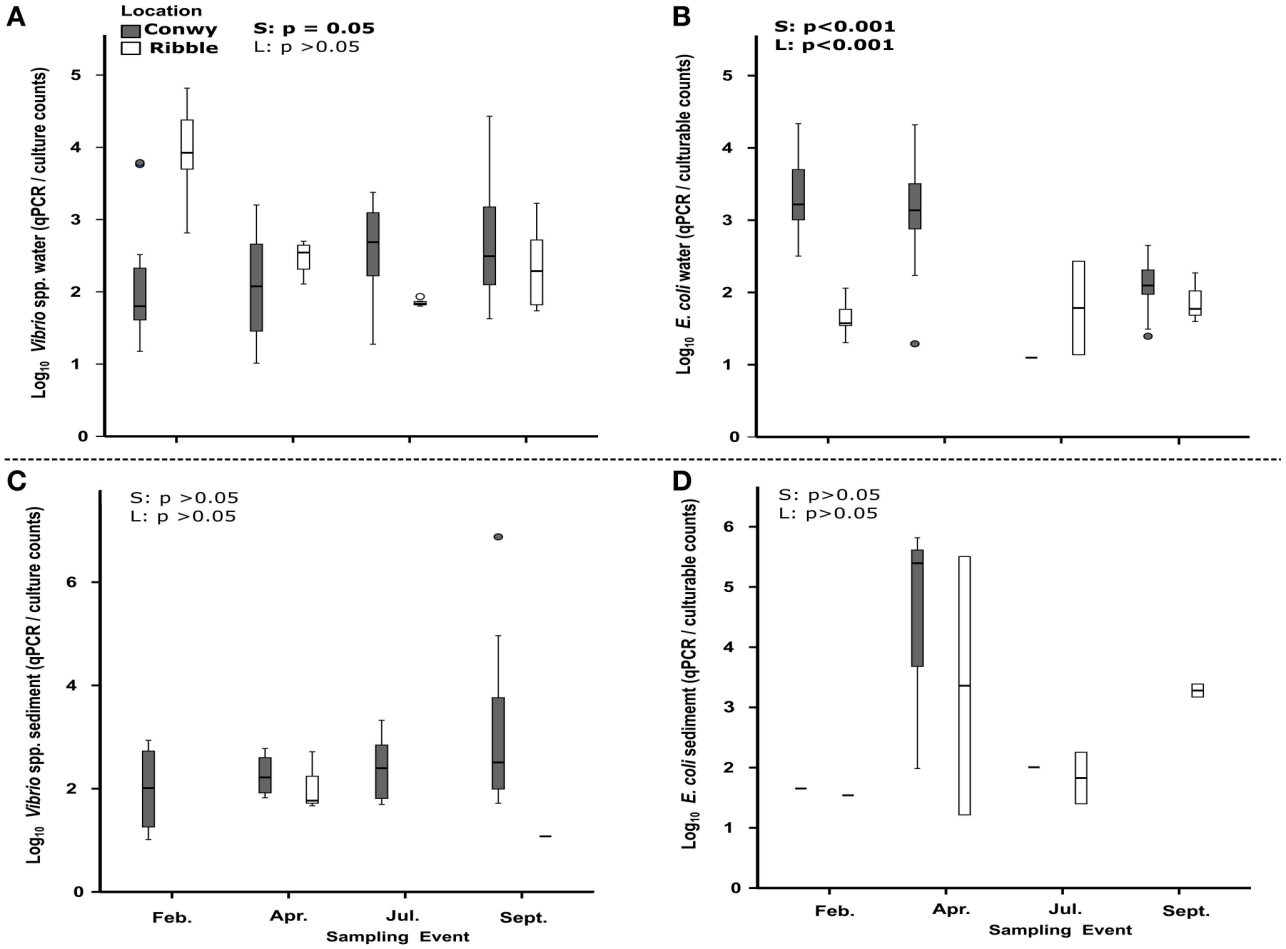
Non-culturability index (qPCR/colony counts) for *Vibrio* **(A,C)** and *E. coli* **(B,D)** in water and sediments. Site data are pooled for each sampling event. Boxes represent 25–75 percentiles and median value (thick black line). Whiskers represent the minimum and maximum data. Outliers and extreme values are plotted as circles and stars respectively. Sites which had a non-culturability index <1 are excluded from analysis. Statistical results of Welch's *t*-test are presented within graphs, with S, effect of season; L, effect of location (Conwy and Ribble). Significant results are in bold.

Culturable fecal indicators (*E. coli*, intestinal enterococci) were recovered from sediment and water from most sites in the Ribble and Conwy estuaries (Figures 3, 4). In this study, results obtained by qPCR were typically more than those from culture-based counting (Figures 3, 4). This is consistent with other studies (Haugland et al., 2005; Sinigalliano et al., 2007; Abdelzaher et al., 2010) which have detected FIOs and pathogens in sediments. Molecular analysis (e.g., qPCR) reveals the presence of microbial genetic material unlike culture-based methods which represent the culturable fraction of the viable bacteria/viruses (De Roda Husman et al., 2009; Ayrapetyan et al., 2014; Jones et al., 2015). A recent study compared epidemiological data, qPCR data and culturable bacterial counts (USEPA, 2012). In the USEPA study, bathing waters with an infection rate of 36 per 1,000 cases, there was a ~13-fold difference between qPCR enterococcus detections and culturable enterococcus providing a useful baseline in waters (USEPA, 2012). It is established that bacteria respond to cues in their environment which impacts culturability and infectivity (Heim et al., 2002; Darcan et al., 2009; Li et al., 2014). In this study we show that the non-culturability ratio can be as high as 4.3 and 4.8 log_10_ qPCR/culturable counts for *E. coli* and *Vibrio* respectively in water in winter 2015, but as low as 1:1 in spring and summer 2015, suggesting a seasonal component to recovery through culture-dependent methods and/or detection by qPCR (Figures 6A,B). We found in the sediment the fold difference was up to 5.8 and 6.9 log_10_ qPCR/culturable counts for *E. coli* and *Vibrio* respectively (Figures 6C,D). Recent work, suggests that intestinal enterococcus retains membrane integrity ~4-fold longer than its culturability, through comparison of propidium monoazide qPCR and culture counts (Gin and Goh, 2013).

The poor specificity of selective growth media can be seen for *Vibrio* and intestinal enterococcus as culture counts often exceeded that of qPCR. One study suggested that 61% of colonies on TCBS media were *Vibrio* spp. (Pfeffer and Oliver, 2003). However, a previous study found that 91% of sequenced isolates from Harlequin *E. coli*/Coliforms media were *E. coli* (Perkins et al., 2014). However, qPCR based methods are also subject to low DNA recovery efficiency and PCR efficiency which are other possible reasons for discrepancies in quantification. *Enterococcus faecalis* and *E. faecium* account for the majority (~80 and ~10%) of human infections, although this amount is highly variable within the human population. Most colonies from enterococcus media sequenced were *E. faecalis* and *E. faecium;* with a successful identification rate of between 74 and 89% (Ferguson et al., 2005) suggesting non-Enterococcus species could account for <26% of observed counts. An additional limitation of culture-based enumeration, is the long incubation periods required for quantification, typically 18–24 h, which prevents their suitability for same-day water quality evaluation (Solo-Gabriele et al., 2015). However, it should be noted that various processing steps are typically required for molecular approaches (qPCR, sequencing), which can result in even longer quantification timescales than culture-based approaches. Rapid diagnostics are under development such as impedance spectroscopy which show some promise for same-day quality evaluation (Jiang et al., 2015).

### Implications for the use of culture based determination of bacterial water and sediment quality

Millions of gastrointestinal and respiratory infections are linked to fecally contaminated coastal and estuarine waters, which are principally viral in origin (Graczyk et al., 2010; Sinigalliano et al., 2010; Halliday and Gast, 2011; Pachepsky and Shelton, 2011; Solo-Gabriele et al., 2015). It is undeniable that culture-based enumeration of bacterial indicators, does not adequately represent the human health risk or the extent of human fecal pollution in bathing/shellfish waters or shellfish destined for human consumption, despite culture-based enumeration being cheap, easy to apply and effective for some scenarios (Gin and Goh, 2013; Pinto et al., 2015; Zhang et al., 2015). Recently, regulators have expressed considerable interest in the use of culture-independent methods (e.g., qPCR or sequencing). Potential barriers include lack of historic datasets on viral pathogens, difficultly comparing historic with new approaches, cost and lack of technical expertise, which together hamper future progress to integrate molecular approaches into routine water quality monitoring (Solo-Gabriele et al., 2015). It is accepted that diverse populations of bacteria including FIOs and pathogens (principally bacterial, viral or protozoan) may survive in soil, sediments and beach sands and an assessment of fecal contamination levels in these matrices are rarely measured in routine surveys (Solo-Gabriele et al., 2015). This is pertinent for sediments which can represent a long-term reservoir of FIOs and pathogens and are often overlooked as a vehicle for infection (Vignaroli et al., 2013). Sediments are subject to forces of resuspension and transport, before subsequently settling out where bacterial indicators/pathogens can persist and accumulate within sediments (Jamieson et al., 2005). Due to contrasting DNA extraction efficiencies between the matrices studied here (i.e., water and sediment), absolute gene/genome copy numbers derived from these samples using qPCR are not directly comparable. However, the general patterns of FIO abundance in the water column and sediment are comparable to previous studies using independent methodologies such as culture counts, which demonstrate that FIOs are more abundant in the sediment than the overlying water column.

The VBNC components of the bacterial community are of concern as they can be metabolically active, potentially pathogenic, but resist culture-based approaches (Lleò et al., 2005). Understanding physicochemical drivers governing the culturability and pathogenicity of bacteria and viruses is of principal concern for the future use of culture-based methods for assessing bathing and shellfish water cleanliness (Solo-Gabriele et al., 2015). This study goes some way toward understanding which physicochemical variables govern FIO abundance and distribution under the dynamic conditions experienced within estuaries. The novel aspects of this study were: (i) the finding that elemental composition of sediments impacts both the abundance/distribution and culturability of bacteria in estuarine environments; (ii) the finding that the abundance of key taxa is temperature-dependent within an annual cycle; (iii) demonstrating clear drivers in physicochemistry and how risk factors (e.g., salinity, temperature and turbidity) influence water quality.

## Conclusions

In conclusion, our study revealed a strong and largely uniform positive relationship between temperature and *Vibrio* concentrations and a broadly negative relationship between *E. coli* and temperature, confirming the elevated levels of FIOs in winter months. The high non-culturability of *Vibrio* and *E. coli* particularly in the Ribble in February 2014 (2.5 log_10_ greater than in Conwy) suggests the culturability of bacteria is linked to prevailing physicochemistry and/or different sources. Further work could identify whether different sources of enteric pollution has variable or similar non-culturability/resuscitation potential. The very high discrepancy between qPCR data and culture-based enumeration particularly in winter challenges the usefulness of FIOs such as *E. coli* as indicators of fecal pollution in shellfish and bathing waters. Additional information provided by conventional and next-generation molecular diagnostics will continue to shed light on the role of physicochemistry on bacterial ecology. Elevated sediment concentrations of bacteria compared to overlying water column (typically 4 log_10_ greater) confirms the role of sediments for the accumulation and persistence of fecal bacteria. Relationships between bacterial indicators/pathogens and physicochemical variables were inconsistent in sediments and no single indicator adequately described the occurrence of all bacterial indicators/pathogens. The concentrations of the elements Zn, Ca, K, and S, organic matter content, porosity, grain size and clay content were significant components describing the variation in bacterial abundance although each variable represented <25% of the variability. Sediments with greater organic matter content and lower porosity harbored greater numbers of non-culturable bacteria in winter providing opportunity for future management. Seasonal trends in bacterial culturability show that combining culture-based and molecular approaches should be considered for the regulation of water quality in designated areas.

## Author contributions

FH, DJ, SM, AA, and JM wrote the manuscript. FH and LP undertook data analysis. VJ, BC, PD, HB provided technical support and reviewed the manuscript. FH, DJ, SM, and AA prepared the manuscript for submission. All authors have approved the final version for submission. All authors had substantial contributions to the conception and design of the work.

### Conflict of interest statement

The authors were commissioned and funded by UK Water Industry Research Limited to carry out this research, which was reported as a chapter presented within a technical report. The funding agency did not influence the content of this paper.
