# Supplementary material for: Physicochemical Factors Influence the Abundance and Culturability of Human Enteric Pathogens and Fecal Indicator Organisms in Estuarine Water and Sediment
Source: Front Microbiol. 2017 Oct 17;8:1996. doi: 10.3389/fmicb.2017.01996 (PMC5650961; doi:10.3389/fmicb.2017.01996)
Supplement: Supplementary file 1 [file Table1.DOC]

Table S1 Selective bacterial growth media used to enumerate target bacterial groups

| **Bacteria target group** | **Media** | **Incubation period** |
| --- | --- | --- |
| *Escherichia coli*/coliform | Harlequin (LabM HAL008) | 24h at 37°C |
| Enterococcus spp. | Slanetz & Bartley (LabM LAB166) | 4h at 37°C subsequently 44h at 45°C |
| Total heterotrophs | R2A (Oxoid, CM0906) | 48h at 25°C |
| Marine heterotrophs | Marine Agar (Deben Diagnostics) | 36h at 25°C |
| Vibrio spp. | Cholera Medium TCBS (Oxoid, CM0333) | 24h at 25°C |
