# Supplementary material for: Physicochemical Factors Influence the Abundance and Culturability of Human Enteric Pathogens and Fecal Indicator Organisms in Estuarine Water and Sediment
Source: Front Microbiol. 2017 Oct 17;8:1996. doi: 10.3389/fmicb.2017.01996 (PMC5650961; doi:10.3389/fmicb.2017.01996)
Supplement: Supplementary file 2 [file Table2.DOC]

Table S2 Target species by qPCR for bacteria: *Campylobacter jejuni*, *Escherichia coli,* *Enterococcus faecalis,* *Enterococcus faecium, Shigella spp, Vibrio spp* and *Salmonella spp.* Target virus by RT-qPCR: Hepatitis A virus, Hepatitis E virus, Norovirus genogroup I and Norovirus genogroup II. The sequence target and gene sequence homology to NCBI database.

| **qPCR target species** | **Sequence target** | **Reference sequence (NCBI database) additional information** | **qPCR or RT-qPCR supplier number Genesig.com** |
| --- | --- | --- | --- |
| **Bacterial DNA** | | |  |
| *Campylobacter jejuni* | CadF gene | CP000814.1, AL111168.1, CP000025.1, AF104303.1, CP000538.1, CP000768.1, AF104302.1, | Path-C.jejuni- |
| *Escherichia coli* | uidA (Glucuronidase) | Broadest detection profile possible whilst remaining specific to the *E.coli* genome | Path-E.coli_spp- |
| *Enterococcus faecalis* | groES heat shock protein | AF335185.1 and AE016830.1 | Path-E.faecalis- |
| *Enterococcus faecium* | groES heat shock protein | AF417582.1 and AY315820.1 | Path-E.faecium- |
| *Shigella* spp | virulence plasmid | pCP301 (VirA) 100% homology with a broad range of clinically relevant reference sequences | Path-Shigella_spp |
| *Vibrio* spp | RNA polymerase alpha subunit (rpoA) gene | 100% homology with a broad range of Vibrio spp sequences | Path-V.cholerae_subsp |
| *Salmonella spp* | invA | 100% homology with a broad range of  Salmonella_invA sequences | Path-Salmonella_invA |
| **Viral RNA** |  |  |  |
| Hepatitis A virus (HAV) | 5’ NCR | Broadest detection profile possible whilst remaining specific to the HAV genome | Path-HAV |
| Hepatitis E virus (HEV) | ORF2 capsid protein gene | Broadest detection profile possible whilst remaining specific to the HEV genome | Path-HEV |
| Norovirus genogroup I | Norovirus GI capsid protein gene | 100% homology with a broad range of clinically relevant reference sequences | Path-Norovirus |
| Norovirus genogroup II | Norovirus GII RNA dependent RNA polymerase gene | 100% homology with a broad range of clinically relevant reference sequences | Path-Norovirus |
