# Supplementary material for: Physicochemical Factors Influence the Abundance and Culturability of Human Enteric Pathogens and Fecal Indicator Organisms in Estuarine Water and Sediment
Source: Front Microbiol. 2017 Oct 17;8:1996. doi: 10.3389/fmicb.2017.01996 (PMC5650961; doi:10.3389/fmicb.2017.01996)
Supplement: Supplementary file 3 [file Table3.DOC]

Table S3 Summary of qPCR on bacterial and viral pathogens for the Conwy and Ribble

| *Target* | Conwy | | Ribble | |
| --- | --- | --- | --- | --- |
|  | Water  (GC/100ml) | Sediment  GC/100g) | Water  (GC100ml) | Sediment  (GC100g) |
| *Salmonella* spp | 10-111  April and June 2015 in Conwy all transects | - | - | - |
| *Campylobacter jeujuni* | <10 GC/100 ml Conwy site 19 in April 2015 | - | - | - |
| *Shigella* spp | - | - | - | - |
| Norovirus GI and GII | - | - | <10 GC GII/100 ml water in February 2015 sites 1 & 2 | - |
| Hepatitis A Virus | - | - | - | - |
| Hepatitis E Virus | - | - | - | - |
| (-) indicates that target not detected in samples by qPCR | | | | |
