# Supplementary material for: Physicochemical Factors Influence the Abundance and Culturability of Human Enteric Pathogens and Fecal Indicator Organisms in Estuarine Water and Sediment
Source: Front Microbiol. 2017 Oct 17;8:1996. doi: 10.3389/fmicb.2017.01996 (PMC5650961; doi:10.3389/fmicb.2017.01996)
Supplement: Supplementary file 4 [file Table4.DOC]

Table S4 Sediment bacterial abundance determined for each site in the Conwy and Ribble estuaries based on average of three repeats and averaged for each transect. Data reported as Log_10_ average and range (minimum and maximum). GC indicates genome copies. ND indicates not detected. All values are expressed per 100g sediment.

|  | **Sept./Oct. 2014** | | | | |  | **Feb. 2015** | | | | | |
| --- | --- | --- | --- | --- | --- | --- | --- | --- | --- | --- | --- | --- |
|  | *E. coli* CFU | *E. coli* GC | *Enterococcus* CFU | *Enterococcus* *faecium* + *faecalis* GC | *Vibrio* CFU | *Vibrio* spp. GC | *E. coli* CFU | *E. coli* GC | *Enterococcus* CFU | *Enterococcus* *faecium* + *faecalis* GC | *Vibrio* CFU | *Vibrio* spp. GC |
|  |
| **Conwy transect 1** | 3.7 (0-4.2) | 2.3 (0-6.9) | 3.8 (0-4.2) | ND | 5.7 (3.7-6.1) | 7.1 (5.9-7.6) | 4.4 (4.1-4.7) | 1.8 (0-5.3) | 3.2 (0-3.5) | ND | 4.8 (4.2-5.2) | 6 (0-6.2) |
| **2** | 5.2 (0-5.8) | ND | 4.8 (0-5.4) | ND | 5.4 (0-6) | 6.2 (0-6.7) | 3.9 (0-4.2) | 2.9 (0-5.9) | 5.5 (3.2-6.1) | ND | 4.9 (4.5-5.2) | 4.5 (0-5.1) |
| **3** | 3.5 (0-3.9) | ND | 4.7 (0-5.2) | 5.4 (0-6) | 5.5 (4.5-5.8) | 6.7 (0-7.3) | 5.1 (5-5.2) | ND | 4.8 (0-5.4) | ND | 5.6 (0-6.2) | ND |
| **4** | 2.7 (0-3.2) | 1.9 (0-5.8) | ND | ND | 4.1 (3.5-4.4) | 7.1 (5.4-7.5) | 5 (0-5.3) | ND | 4 (0-4.5) | 4.7 (0-5.1) | 5.9 (3.9-6.3) | 6.8 (0-7.2) |
| **5** | ND | ND | ND | ND | 5 (5-5) | 6.4 (6.4-6.4) | ND | ND | ND | ND | 4.9 (4.9-4.9) | ND |
| **Ribble sites 1-2** | 4.8 (4.3-5) | 7.9 (7.7-8.2) | 2.9 (0-3.2) | ND | 7.1 (6.9-7.3) | 5 (0-5.3) | 3.6 (0-3.9) | ND | 4.7 (4.3-4.9) | ND | 7 (6.7-7.2) | ND |
| **Ribble sites 3-8** | 4.9 (0-5.7) | 2.1 (0-6.7) | 4.6 (0-5.4) | ND | 6.6 (6-7.3) | 6.4 (0-7.1) | 3.9 (0-4.4) | 1 (0-5.9) | 4.2 (0-4.7) | 4.4 (0-5.2) | 6.5 (5.8-6.9) | 5.5 (0-6) |

|  | **Apr. 2015** | | | | | | **Jun./Jul. 2015** | | | | | |
| --- | --- | --- | --- | --- | --- | --- | --- | --- | --- | --- | --- | --- |
| Conwy Transect | *E. coli* CFU | *E. coli* GC | *Enterococcus* CFU | *Enterococcus* *faecium* + *faecalis* GC | *Vibrio* CFU | *Vibrio* spp. GC | *E. coli* CFU | *E. coli* GC | *Enterococcus* CFU | *Enterococcus* *faecium* + *faecalis* GC | *Vibrio* CFU | *Vibrio* spp. GC |
| Ribble site |
| **Conwy transect 1** | 2.7 (0-3.2) | 5.3 (5.2-5.4) | 3.7 (0-4.2) | ND | 5.4 (3.9-5.9) | 6.3 (5.4-6.7) | 3.8 (0-4.2) | 2.1 (0-6.2) | 3.9 (0-4.4) | ND | 6 (4.9-6.2) | 7 (5.4-7.4) |
| **2** | ND | ND | ND | ND | 4.5 (3.9-4.8) | 4.9 (0-5.5) | 2.6 (0-3.2) | ND | 2.6 (0-3.2) | ND | 5.9 (5.6-6) | 7.1 (0-7.6) |
| **3** | 5.6 (3.8-5.9) | 2.9 (0-6) | 4.6 (0-5.1) | 4.4 (0-5) | 5.3 (0-5.7) | 7 (0-7.5) | 4.3 (0-4.8) | ND | 3.8 (0-4.2) | 5.2 (0-5.8) | 5.4 (4.2-5.8) | 7.3 (0-7.9) |
| **4** | ND | ND | 2.7 (0-3.2) | ND | 5 (0-5.5) | 5.9 (0-6.3) | ND | ND | 3 (0-3.2) | ND | 4.7 (0-5.1) | 6.1 (0-6.4) |
| **5** | ND | ND | ND | ND | 5.5 (5.5-5.5) | 6.2 (6.2-6.2) | ND | ND | 0 (0-0) | ND | 4.9 (4.9-4.9) | 6.6 (6.6-6.6) |
| **Ribble sites 1-2** | 3.8 (0-4.1) | 5.3 (5.2-5.5) | 4 (3.5-4.2) | 5.5 (0-5.8) | 7 (6.4-7.2) | 6.2 (6.1-6.2) | 4.6 (4.1-4.8) | 3.1 (0-6.2) | 4.2 (3.9-4.4) | ND | 5.6 (5.5-5.7) | 7.1 (6.8-7.3) |
| **Ribble sites 3-8** | 3.9 (0-4.6) | ND | 3.1 (0-3.7) | 6 (0-6.8) | 6.8 (6.3-7.3) | 5.6 (0-6.3) | 3.6 (0-3.9) | 1 (0-5.9) | 4.2 (3.5-4.7) | ND | 5 (4.6-5.2) | 5.8 (0-6.5) |
