# Supplementary material for: Physicochemical Factors Influence the Abundance and Culturability of Human Enteric Pathogens and Fecal Indicator Organisms in Estuarine Water and Sediment
Source: Front Microbiol. 2017 Oct 17;8:1996. doi: 10.3389/fmicb.2017.01996 (PMC5650961; doi:10.3389/fmicb.2017.01996)
Supplement: Supplementary file 5 [file Table5.DOC]

**Table S5** Distance Based Linear Models (DistLM) marginal tests. Response variables are log_10_ bacterial abundance in water (CFU/100ml and GC /100ml) and sediment (CFU/100g and GC /100g) based on pooled sample event data. Non-culturability index between bacterial target counts log_10_ (qPCR/CFU) in water and sediment. Predictor variables for water include temperature, salinity, pH and turbidity. Predictors variables for sediment include mean grain size, organic matter content, % clay, Al, K, Fe and Zn. Model outputs including R^2^, Sums of Squares (SS), degrees of freedom (df) are presented, values p<0.05 are deemed significant and are in bold for marginal test.

| Bacterial group/variable | R^2^ | SS | Pseudo-F | p | Proportion of variation (%) | Residual df |
| --- | --- | --- | --- | --- | --- | --- |
| Response variable bacterial abundance water | | | | | | |
| Salinity | 0.16 | 9269 | 20.3 | **<0.001** | 16.0 | 106 |
| Temperature | 0.19 | 1792 | 4.0 | **<0.001** | 3.8 | 105 |
| pH | 0.31 | 6994 | 18.3 | **<0.001** | 12.6 | 104 |
| Turbidity | 0.37 | 3575 | 10.2 | **<0.001** | 6.1 | 103 |
| Response variable log_10_ (Non-culturability index qPCR/CFU) water | | | | | | |
| Salinity | 0.10 | 11190 | 12.0 | **0.001** | 10.0 | 106 |
| Temperature | 0.04 | 4404 | 4.4 | **0.03** | 14.2 | 105 |
| Response variable bacterial abundance sediment | | | | | | |
| Mean grain size | 0.09 | 12231 | 9.5 | **<0.001** | 9.6 | 90 |
| Salinity | 0.14 | 5701 | 4.6 | **<0.001** | 4.5 | 89 |
| K | 0.17 | 3933 | 3.3 | **0.02** | 3.1 | 88 |
| Bulk density | 0.20 | 3564 | 3.0 | **0.03** | 2.8 | 87 |
| Response variable Non-culturability index (qPCR/CFU) sediment | | | | | | |
| Salinity | 0.05 | 15432 | 5.5 | **0.002** | 5.8 | 90 |
| Zn | 0.10 | 11398 | 4.3 | **0.003** | 4.3 | 89 |
| Organic matter content (%) | 0.13 | 8781 | 3.4 | **0.015** | 3.3 | 88 |
| S | 0.16 | 7587 | 2.9 | **0.031** | 2.8 | 87 |
| Mean grain size | 0.18 | 5622 | 2.2 | **0.05** | 2.1 | 86 |
| Porosity (%) | 0.21 | 6017 | 2.4 | 0.06 | 2.3 | 85 |
