# Supplementary material for: Physicochemical Factors Influence the Abundance and Culturability of Human Enteric Pathogens and Fecal Indicator Organisms in Estuarine Water and Sediment
Source: Front Microbiol. 2017 Oct 17;8:1996. doi: 10.3389/fmicb.2017.01996 (PMC5650961; doi:10.3389/fmicb.2017.01996)
Supplement: Supplementary file 6 [file Table6.DOC]

**Table S6** Chemical composition of sediment samples from Conwy and Ribble estuaries average and range (pooled sampling events). All values are mg kg^-1^.

| Element | Al | Ca | Cu | Fe | K | P | S | Zn |
| --- | --- | --- | --- | --- | --- | --- | --- | --- |
| Conwy site | | | | | | | | |
| 1 | 8443 (3743-13736) | 5731 (1108-12303) | 4 (1-7) | 6562 (4082-8351) | 3258 (2334-4262) | 205 (72-363) | 435 (53-799) | 74 (65-92) |
| 2 | 9662 (4861-14901) | 7571 (763-17850) | 5 (2-10) | 8212 (5507-13408) | 4125 (3249-6044) | 229 (90-414) | 526 (38-906) | 109 (65-154) |
| 3 | 6718 (4002-12242) | 4278 (941-11233) | 4 (2-11) | 6488 (3706-12199) | 3046 (1664-5264) | 146 (64-331) | 441 (146-899) | 87 (62-110) |
| 4 | 5060 (4148-6033) | 1882 (861-3056) | 2 (2-3) | 4856 (3310-5466) | 3258 (2731-4273) | 84 (0-196) | 369 (44-998) | 75 (61-100) |
| 5 | 3349 (806-5472) | 2137 (263-3315) | 2 (0-4) | 3233 (805-5308) | 2051 (416-3826) | 75 (0-124) | 137 (79-181) | 44 (18-65) |
| 6 | 4316 (2896-6978) | 1489 (907-2680) | 1 (1-2) | 4617 (2761-6420) | 3008 (1582-5691) | 75 (48-123) | 280 (56-556) | 68 (34-105) |
| 7 | 17946 (2061-47443) | 10730 (915-27834) | 18 (0-40) | 16725 (2256-41483) | 7811 (1256-17812) | 483 (0-1257) | 1016 (252-2771) | 144 (28-366) |
| 8 | 7909 (1881-13786) | 5807 (1781-12281) | 4 (1-7) | 7251 (2219-12581) | 3754 (1336-6639) | 182 (37-473) | 301 (244-369) | 67 (22-116) |
| 9 | 4093 (0-7661) | 3990 (0-8836) | 2 (0-6) | 3773 (0-6722) | 2148 (0-3868) | 84 (0-195) | 275 (0-598) | 41 (0-71) |
| 10 | 2069 (0-6729) | 10896 (0-31183) | 0 (0-2) | 2500 (0-8570) | 1376 (0-3308) | 243 (0-487) | 235 (0-505) | 24 (0-47) |
| 11 | 12467 (0-30541) | 12834 (2114-24048) | 12 (2-24) | 15691 (5466-29988) | 7722 (4273-13372) | 389 (196-618) | 926 (287-1491) | 135 (83-221) |
| 12 | 7924 (1679-16091) | 10021 (1723-15015) | 5 (1-12) | 6418 (1376-13082) | 4586 (814-8117) | 191 (26-347) | 709 (107-1980) | 61 (17-113) |
| 13 | 4096 (0-8642) | 11400 (10532-12268) | 5 (3-7) | 3296 (0-6732) | 3797 (3623-3970) | 164 (65-263) | 681 (76-1285) | 49 (31-67) |
| 14 | 3150 (996-6439) | 4013 (1641-8239) | 1 (0-3) | 2813 (1007-5647) | 1881 (749-3653) | 18 (0-36) | 182 (87-356) | 53 (6-141) |
| 19 | 2950 (1514-4215) | 17406 (3794-46439) | 0 (0-1) | 2878 (2002-3996) | 1793 (976-2630) | 182 (55-387) | 255 (125-418) | 23 (9-31) |
| Ribble site | | | | | | | | |
| 1 | 4316 (1663-7808) | 11400 (6899-18866) | 2 (1-4) | 4083 (2449-7098) | 2707 (1635-4186) | 174 (109-235) | 236 (151-329) | 24 (13-41) |
| 2 | 8038 (2833-14979) | 12557 (5618-19052) | 7 (2-12) | 7458 (2716-12746) | 3724 (1648-6503) | 258 (98-491) | 383 (177-658) | 49 (16-84) |
| 3 | 4501 (1742-8229) | 11835 (6175-14982) | 2 (1-4) | 4463 (2338-6659) | 3234 (1557-5590) | 155 (48-250) | 222 (186-294) | 22 (12-31) |
| 4 | 2963 (709-7909) | 9948 (6828-18037) | 2 (0-5) | 3349 (1264-8355) | 1907 (552-4987) | 104 (74-139) | 180 (118-275) | 18 (0-55) |
| 5 | 3697 (2569-5021) | 11250 (9124-14271) | 2 (0-4) | 4076 (3464-4741) | 2445 (1992-3617) | 157 (44-261) | 279 (158-503) | 22 (16-28) |
| 6 | 5381 (1045-12663) | 11890 (8181-16073) | 6 (0-18) | 5947 (1559-14594) | 3197 (973-5624) | 184 (52-449) | 399 (193-791) | 39 (9-106) |
| 7 | 2947 (1943-3711) | 11759 (6401-19931) | 2 (0-4) | 3053 (2027-4260) | 1838 (1484-2490) | 177 (37-350) | 322 (98-654) | 20 (13-39) |
| 8 | 6368 (1803-12402) | 12848 (7325-20571) | 5 (2-11) | 6113 (2664-10759) | 3454 (1728-5469) | 169 (42-401) | 302 (76-680) | 34 (18-69) |
| Sediment could not be collected at Conwy sites 15-18 | | | | | | | | |
