# Supplementary material for: Physicochemical Factors Influence the Abundance and Culturability of Human Enteric Pathogens and Fecal Indicator Organisms in Estuarine Water and Sediment
Source: Front Microbiol. 2017 Oct 17;8:1996. doi: 10.3389/fmicb.2017.01996 (PMC5650961; doi:10.3389/fmicb.2017.01996)
Supplement: Supplementary file 7 [file DataSheet1.DOC]

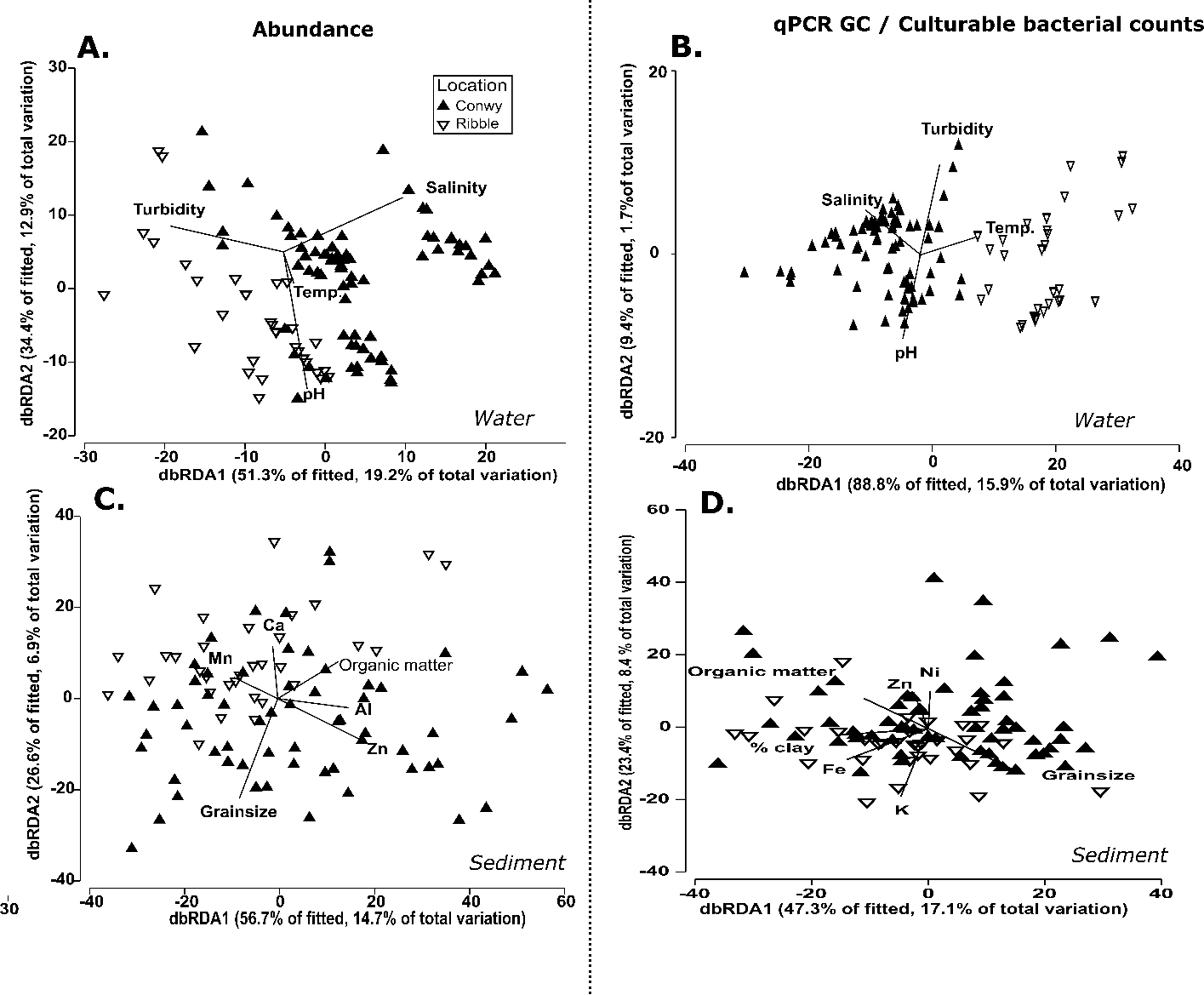


**Figure S1.** Distance Based Redundancy Analysis (dBRDA) of parsimonious models across locations and sampling events. The abundance of bacteria (*Escherichia coli*, *Enterococcus faecalis*, *E. faecium, Vibrio spp., Campylobacter jeujuni, Shigella spp., Salmonella spp. and coliforms*) was modelled in A. water, B. sediment. The culturability fold difference between bacterial target counts log10(qPCR/CFU) (groups included *Escherichia coli*, Enterococcus, Vibrio) was modelled in C. water D. sediment. In all cases the physicochemical variables are superimposed as a correlations biplot with only significant correlations with R^2^ >0.5 included.
